# Metformin delays neurological symptom onset in a mouse model of neuronal complex I deficiency

**DOI:** 10.1172/jci.insight.141183

**Published:** 2020-11-05

**Authors:** Susana Peralta, Milena Pinto, Tania Arguello, Sofia Garcia, Francisca Diaz, Carlos T. Moraes

**Affiliations:** 1 Department of Neurology and; 2 Department of Cell Biology, Miller School of Medicine, University of Miami, Miami, Florida, USA.

**Keywords:** Genetics, Mitochondria, Mouse models

## Abstract

Complex I (also known as NADH-ubiquinone oxidoreductase) deficiency is the most frequent mitochondrial disorder present in childhood. NADH-ubiquinone oxidoreductase iron-sulfur protein 3 (NDUFS3) is a catalytic subunit of the mitochondrial complex I; NDUFS3 is conserved from bacteria and essential for complex I function. Mutations affecting complex I, including in the *Ndufs3* gene, cause fatal neurodegenerative diseases, such as Leigh syndrome. No treatment is available for these conditions. We developed and performed a detailed molecular characterization of a neuron-specific *Ndufs3* conditional KO mouse model. We showed that deletion of *Ndufs3* in forebrain neurons reduced complex I activity, altered brain energy metabolism, and increased locomotor activity with impaired motor coordination, balance, and stereotyped behavior. Metabolomics analyses showed an increase of glycolysis intermediates, suggesting an adaptive response to the complex I defect. Administration of metformin to these mice delayed the onset of the neurological symptoms but not of neuronal loss. This improvement was likely related to enhancement of glucose uptake and utilization, which are known effects of metformin in the brain. Despite reports that metformin inhibits complex I activity, our findings did not show worsening a complex I defect nor increases in lactic acid, suggesting that metformin should be further evaluated for use in patients with mitochondrial encephalopathies.

## Abstract 2

Metformin delays onset of mitochondrial encephalopathy in a CNS model of mitochondrial oxidative phosphorylation defect.

## Graphical abstract

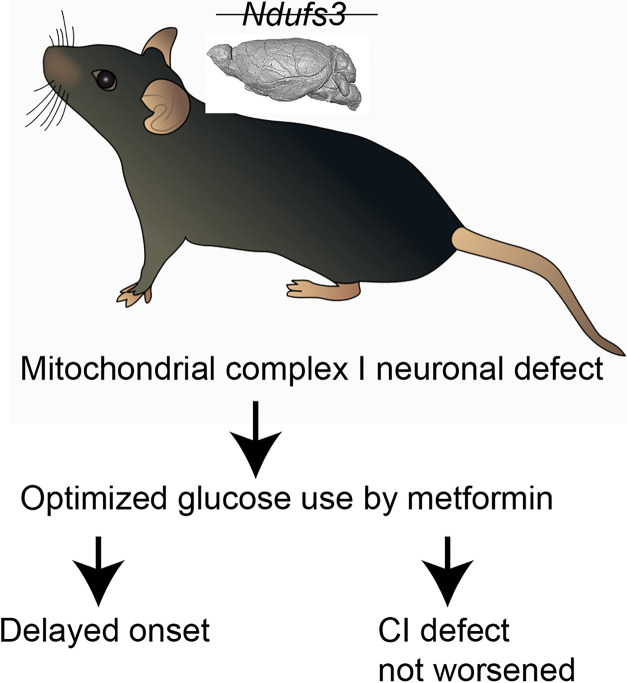

## Introduction

Complex I (also known as NADH-ubiquinone oxidoreductase), the first complex of the electron transport chain, catalyzes electron transfer to ubiquinone from the oxidation of NADH (1). This electron transfer is coupled to the pumping of protons across the mitochondrial inner membrane to help generate the electrochemical gradient needed for the synthesis of ATP through CV or ATP synthase (2). Mammalian CI, with a molecular weight of approximately 980 kDa, is the largest oxidative phosphorylation (OXPHOS) complex; it is composed of 45 subunits encoded by both mitochondrial DNA (mtDNA) and the nuclear DNA (nDNA) (3). CI deficiency is the most common cause of respiratory chain defects in childhood (4). CI deficiency can result from mutations in either mtDNA- or nDNA-encoded subunits or assembly factors (5). However, mutations in nuclear genes coding for CI subunits are the most frequent cause of CI deficiencies by far (6, 7). Most affected children with CI deficiency are diagnosed with Leigh syndrome (LS), which is characterized by psychomotor retardation, myopathy, dyspnea, cerebellar ataxia, lactic acidosis, and progressive encephalopathy primarily in the brainstem and basal ganglia (8–10). Other CI disorders associated with infantile onset include leukoencephalopathy (11, 12); fatal infantile lactic acidosis (13, 14); mitochondrial encephalomyopathy, lactic acidosis, and stroke-like episodes (15); and hypertrophic cardiomyopathy (16–18). CI defects may also cause Leber’s hereditary optic neuropathy, which affects predominantly retinal ganglion cells (19).

NADH-ubiquinone oxidoreductase iron-sulfur protein 3 (NDUFS3) is one of the nDNA-encoded subunits required for CI formation and activity (20). Several patients with missense mutations in the *NDUFS3* gene had severe phenotypes and a fatal outcome within a few years after onset. Compound heterozygosity for *Ndufs3* mutations (T145I and R199W) has been reported in 1 patient with late-onset Leigh syndrome, optic atrophy, and CI deficiency (21), whereas the compound heterozygosity for the mutations (R140W and R199W) has been associated with early-onset Leigh syndrome and severe reduction in CI levels (23, 24). In homozygosity, the R199W mutation caused developmental delay, encephalopathy, myopathy, and lactic acidosis (22). In this work, we generated a CI neuron-specific conditional KO (nKO) mouse model by deleting the *Ndufs3* gene specifically in forebrain neurons. The model exhibits a fatal encephalopathy and recapitulates some of the features observed in patients with CI defects, such as ataxia, gliosis, neuronal cell death, weight loss, and reduced survival.

Unfortunately, there is no treatment for neurodegenerative diseases associated with CI deficiency. Therapeutic intervention based on altering the cellular metabolism, such as administration of a high-fat diet (25) and inhibition of mTOR (26), both of which affect carbohydrate metabolism, delayed the neurological symptoms in 2 mouse models of CI encephalopathies. Metformin (also known as 1,1-dimethylbiguanide) is a potent regulator of cellular metabolism and one of the most common hypoglycemic drugs used to treat type 2 diabetes in humans because of its beneficial effects and safety. In control mice, metformin increased insulin sensitivity and reduced both oxidative damage accumulation and chronic inflammation (27). In mammalian models of Alzheimer’s disease (28), multiple sclerosis (29), and Huntington’s disease (30) metformin prevented neurodegeneration. To investigate whether metformin can be used as an effective strategy to ameliorate a mitochondrial encephalopathy, we administered metformin to the neuron-specific *Ndufs3* conditional KO mice (*Ndufs3* nKO) mice and assessed its effects in the onset and progression of disease. Administration of metformin to the *Ndufs3* nKO mice did not aggravate the existing CI deficiency and delayed the onset of the neurological symptoms.

## Results

### Creation and characterization of a mouse model of CI deficiency — neuron-specific Ndufs3 conditional KO mice.

**Figure 1:**
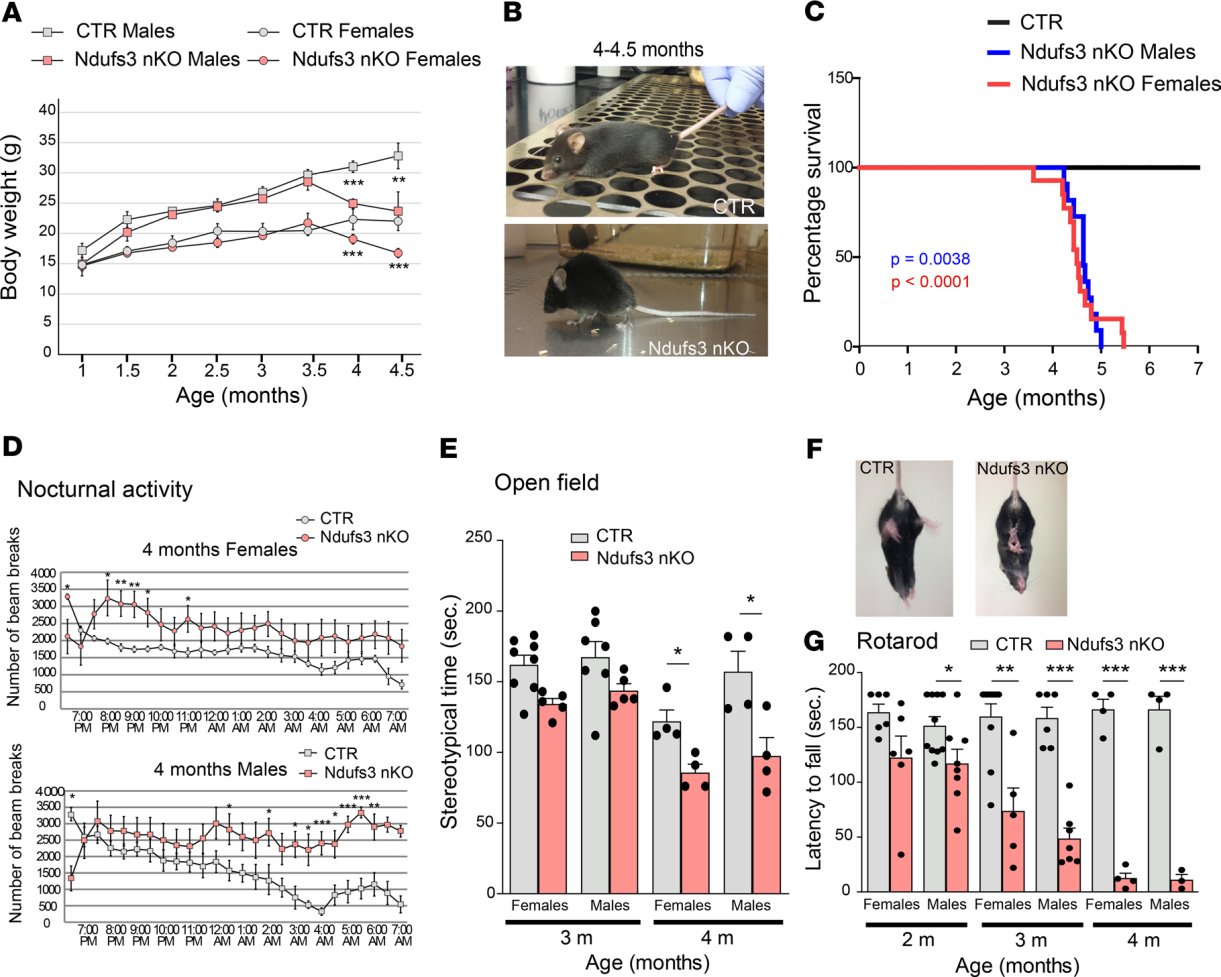
Characterization of *Ndufs3* nKO mice. (**A**) Body weight comparison over time of *Ndufs3* nKO male mice (pink squares; *n* = 6), age-matched control male mice (gray squares; *n* = 7), *Ndufs3* nKO female mice (pink circles; *n* = 6), and age-matched control female mice (gray circles; *n* = 6). *P* values were calculated by Student’s *t* test. (**B**) Representative image of a 4- to 4.5-month-old *Ndfus3* nKO mouse and a control (CTR) littermate, showing decreased body weight and kyphosis. (**C**) Survival curve of *Ndufs3* nKO female mice (red line) and male mice (blue line). *P* values were calculated using log-rank (Mantel-Cox) test. *P* = 0.0038 for male mice (*n* = 15); *P* < 0.0001 for female mice (*n* = 14). Survival was reduced in the *Ndufs3* nKO mice: all Ndfus3 nKO male mice died before 5 months of age, all Ndfus3 nKO female mice died before 6 months of age. (**D**) Nocturnal ambulatory activity of 4-month-old *Ndufs3* nKO female mice (pink circles; *n* = 11), control female mice (gray circles *n* = 14), *Ndufs3* nKO male mice (pink squares; *n* = 7), control male mice (gray squares; *n* = 6). *P* values were calculated by Student’s *t* test. (**E**) Stereotypical time of 3- and 4-month-old *Ndufs3* nKO and control male and female mice (*n* = 4–8/group). (**F**) Representative image of a tail suspension test of 4-month-old *Ndfus3* nKO and control littermate. *Ndfus3* nKO mice clasped the 4 limbs, while control mice extended the legs in preparation for the contact. (**G**) Rotarod performed by *Ndufs3* nKO and control mice at 2, 3, and 4 months of age (*n* = 4–10). Data are represented as mean ± SEM. *P* values were calculated by Student’s *t* test to determine the level of statistical difference. *P < 0.05, **P < 0.01, ***P < 0.0001.

To KO *Ndufs3* in neurons, mice homozygous for the floxed *Ndufs3* gene (*Ndufs3*^fl/fl^, Supplemental Figure 1, A–C; supplemental material available online with this article; https://doi.org/10.1172/jci.insight.141183DS1) were bred with transgenic mice expressing Cre recombinase under the control of the α subunit of the calcium/calmodulin-dependent protein kinase II (CaMKIIa) promoter (31). The CaMKIIa gene is expressed in excitatory neurons in the forebrain, predominantly in cortex and hippocampus. Its expression starts at E18.5 and reaches full activity by P60. *Ndufs3*^fl/fl^-CaMKIIa-Cre^+/–^ mice (neuron-specific Ndufs3 conditional KO mice [herein referred to as *Ndufs3 nKO* mice]) were used as experimental animals and were compared with either *Ndufs3*^fl/fl^-CaMKIIa-Cre^–/–^ or *Ndufs3*^F/W^-CaMKIIa-Cre^–/–^ control animals (Supplemental Figure 1C). The mice were genotyped as illustrated in Supplemental Figure 1B, and the excision of exons 3 and 4 was confirmed by PCR (Supplemental Figure 1, D and E). *Ndufs3* nKO mice were born at Mendelian ratios; during the first 3.5 months of life, they were indistinguishable from control mice with regard to physical appearance and body weight (Figure 1A). Starting at 4 months of age, *Ndufs3* nKO mice showed a dramatic reduction in body weight (both male and female mice) that progressed rapidly (Figure 1A). Four-month-old *Ndufs3* nKO mice stopped grooming and showed kyphosis (Figure 1B) and ataxia (Supplemental Video 1). In addition, *Ndufs3* nKO mice showed hypersensitivity to external stimuli (e.g., when being handled by the investigator and when exploring a new environment, Supplemental Video 1). With time, *Ndufs3* nKO mice lost motor coordination and balance and had catatonic/lethargic episodes (Supplemental Video 1). *Ndufs3* nKO mice (male and female mice) died at between 4.5 and 5.5 months of age (Figure 1C).

To detect the onset of a motor phenotype in these mice, we used several behavioral assays, including locomotor activity cage, open-field test, and rotarod. *Ndufs3* nKO mice at 2 and 3 months of age (male and female mice) showed normal nocturnal locomotor activity (active period) (Supplemental Figure 2) and normal exploratory activity (Figure 1E). Starting at 4 months of age, they presented increased nocturnal locomotor activity (Figure 1D) and decreased stereotypical movement time (Figure 1E). All 4-month-old *Ndufs3* nKO male and female mice tested presented a strong limb clasping phenotype when suspended by the tail (Figure 1F). Moreover, motor coordination measured with rotarod was significantly decreased at 2 months of age in *Ndufs3* nKO male mice and starting at 3 months of age in female mice (Figure 1G). For both sexes, motor coordination worsened with time (Figure 1G).

### Lack of NDUFS3 expression is associated with isolated CI deficiency.

**Figure 2:**
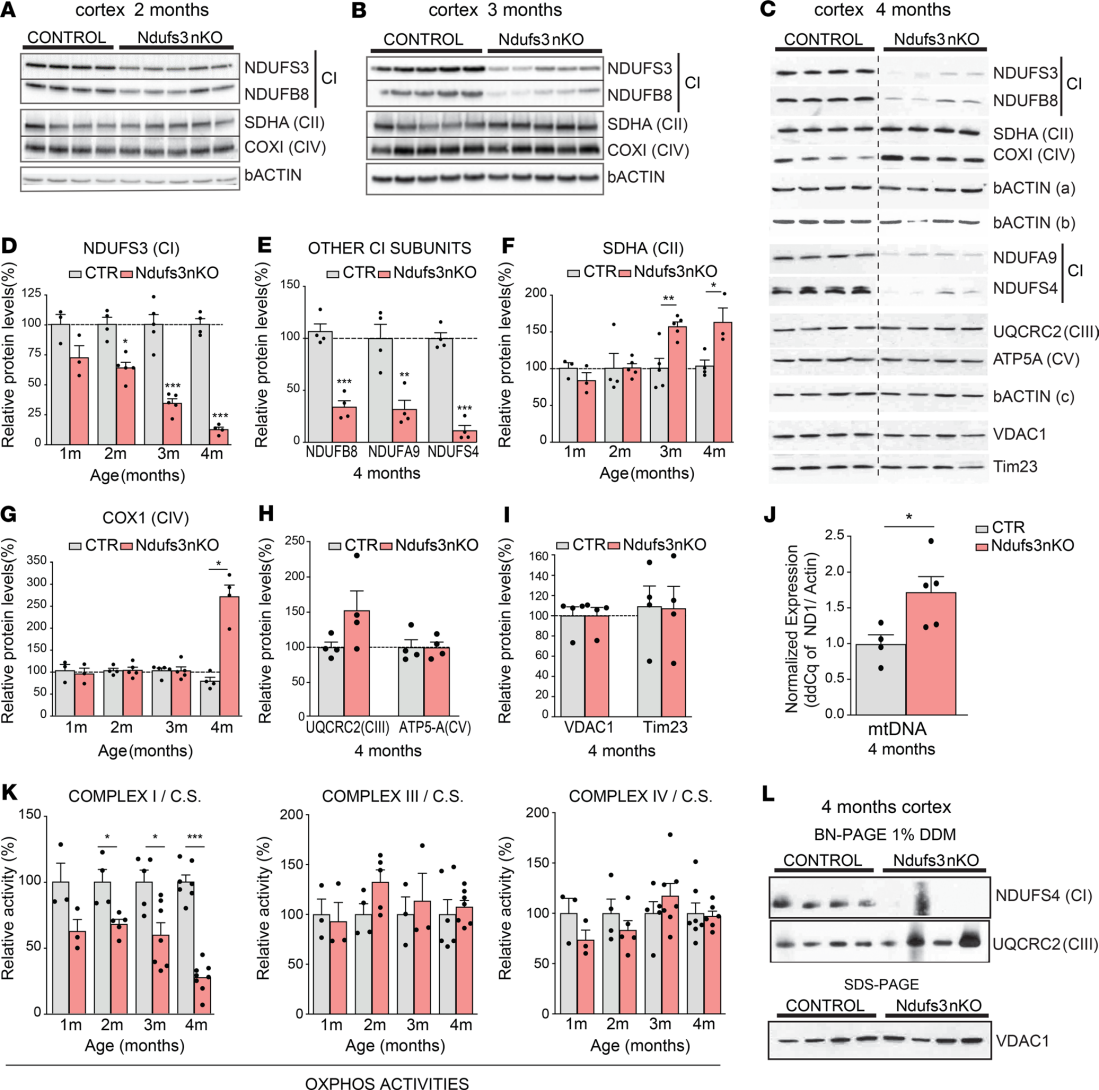
NDUFS3 expression and complex I deficiency. (**A**–**C**) Western blots of cortex homogenates of control (CTR) and *Ndufs3* nKO male mice at different ages (2, 3, and 4 months, respectively) using antibodies against NDUFS3, NDUFB8, NDUFA9, and NDUFS4 (complex I subunits); SDHA (complex II subunit); UQCRC2 (complex III subunit); COX1 (complex IV subunit); ATP5A (complex V subunit); and VDAC1, Tim23, and β-actin [β-actin (a) for NDUFS3, SDHA, and NDUFS4; β-actin (b) for NDUFB8 and COXI; β-actin (c) for NDUFA9 and ATP5A; and β-actin (d) for UQCRC2, VDAC1, and Tim23]. The dashed line in **C** indicates that the gel image was cropped to remove lanes not relevant to the analysis. (**D**–**I**) Quantification of the Western blots in **A**–**C**. Data are represented as mean ± SEM (*n* = 4–5/group). *P* values were determined by Student’s *t* test. (**J**) mtDNA levels measured by RT-PCR in DNA extracted from cortices of 4-month-old control and *Ndufs3* nKO male mice (*n* = 4–5/group). (**K**) Spectrophotometric complex I/citrate synthase, complex III/citrate synthase, and complex IV/citrate synthase activity ratios were measured in cortex homogenates from 1-, 2-, 3-, and 4-month-old male mice. Complex I activity in *Ndufs3* nKO animals was decreased in comparison with that in control mice. Data are represented as mean ± SEM (*n* = 3–8/group). *P* values were determined by Student’s *t* test. (**L**) Steady-state levels of complex I and III measured by BN-PAGE in homogenates from cortices of control and *Ndufs3* nKO mice at 4 months of age using antibodies against NDUFS4 (complex I) and UQCRC2 (complex III) subunits and relative mitochondrial content (Western blot of the same homogenates using antibody against VDAC1). *P < 0.05, **P < 0.01, ***P < 0.001

To verify the lack of NDUFS3 protein in cortical neurons, we performed Western blot of cortex homogenates of 1-, 2-, 3-, and 4-month-old control and *Ndufs3* nKO mice. The level of NDUFS3 in cortices from 1-month-old *Ndufs3* nKO male mice was comparable to that in controls (Supplemental Figure 3, A and B) but decreased progressively with time starting at 2 months (Figure 2, A, B, and D). In cortices of 4-month-old *Ndufs3* nKO mice, NDUFS3 was markedly reduced to approximately 10% of the control levels (Figure 2, C and D). We also analyzed the steady-state levels of other CI subunits located in different modules of the fully assembled complex, such as NDUFB8, NDUFA9, and NDUFS4, and found them dramatically decreased at 4 months of age (Figure 2, C and E). To determine if the decrease of the CI subunits affected the other OXPHOS complexes, we analyzed the steady-state levels of a representative subunit of each complex. We detected increased CII subunit (SDHA) in the samples of 3- and 4-month-old *Ndufs3* nKO mice (Figure 2, C and F). A similar increase was observed in the levels of the CIV subunit COX1 at 4 months but not at earlier ages (Figure 2, C and G). No differences were found in the levels of the CIII subunit UQCRC2 nor CV subunit ATP5-α (Figure 2, C and H). To determine if the increased levels of CII and CIV subunits were due to augmented mitochondrial mass, we measured the steady-state levels of 2 additional mitochondrial membrane proteins, VDAC1 and Tim23, which were not altered (Figure 2, C and I). mtDNA levels instead were increased in cortices from *Ndufs3* nKO mice compared with those from control littermates (1.7-fold of the controls, Figure 2J), which may have contributed to the increased levels of the mtDNA-encoded subunit COX1.

We measured OXPHOS complex enzymatic activity in cortical homogenates of animals at different ages. Rotenone-sensitive CI activity of *Ndufs3* nKO mice was reduced to approximately 67% of that of controls at 2 months of age (Figure 2K). This significant decrease in CI activity coincided with the onset of the phenotype (Figure 1G). CI activity in *Ndufs3* nKO cortices remained reduced to similar levels at 3 months of age (~60%) but dropped sharply to approximately 25% at the age of 4 months (Figure 2K). Given that in the homogenates we have glial and endothelial cells in which the *Ndufs3* gene has not been knocked out, the actual decrease in neuronal CI activity should be higher. CIII and CIV activities remained unchanged in the cortices of 1-, 2-, 3-, and 4-month-old *Ndufs3* nKO mice (Figure 2K). Likewise, citrate synthase activity in *Ndufs3* nKO cortex homogenates was similar to that of controls at all ages tested (Supplemental Figure 3C).

NDUFS3 forms a subassembly complex together with NDUFS2, NDUFS7, and NDUFS8 during the early assembly stages of CI (32, 33). Therefore, we expected that loss of NDUFS3 would impair CI assembly. Indeed, by Blue Native–PAGE (BN-PAGE) probing of the catalytic subunit NDUFS4, we found a dramatic decrease in the levels of the fully assembled CI in the samples from 4-month-old *Ndufs3* nKO mice (Figure 2L). As expected, CI in-gel activity was also reduced in *Ndufs3* nKO cortex homogenates (Supplemental Figure 3D).

### Glycolysis and pentose phosphate pathway metabolites were altered in Ndufs3 KO brains.

**Figure 3:**
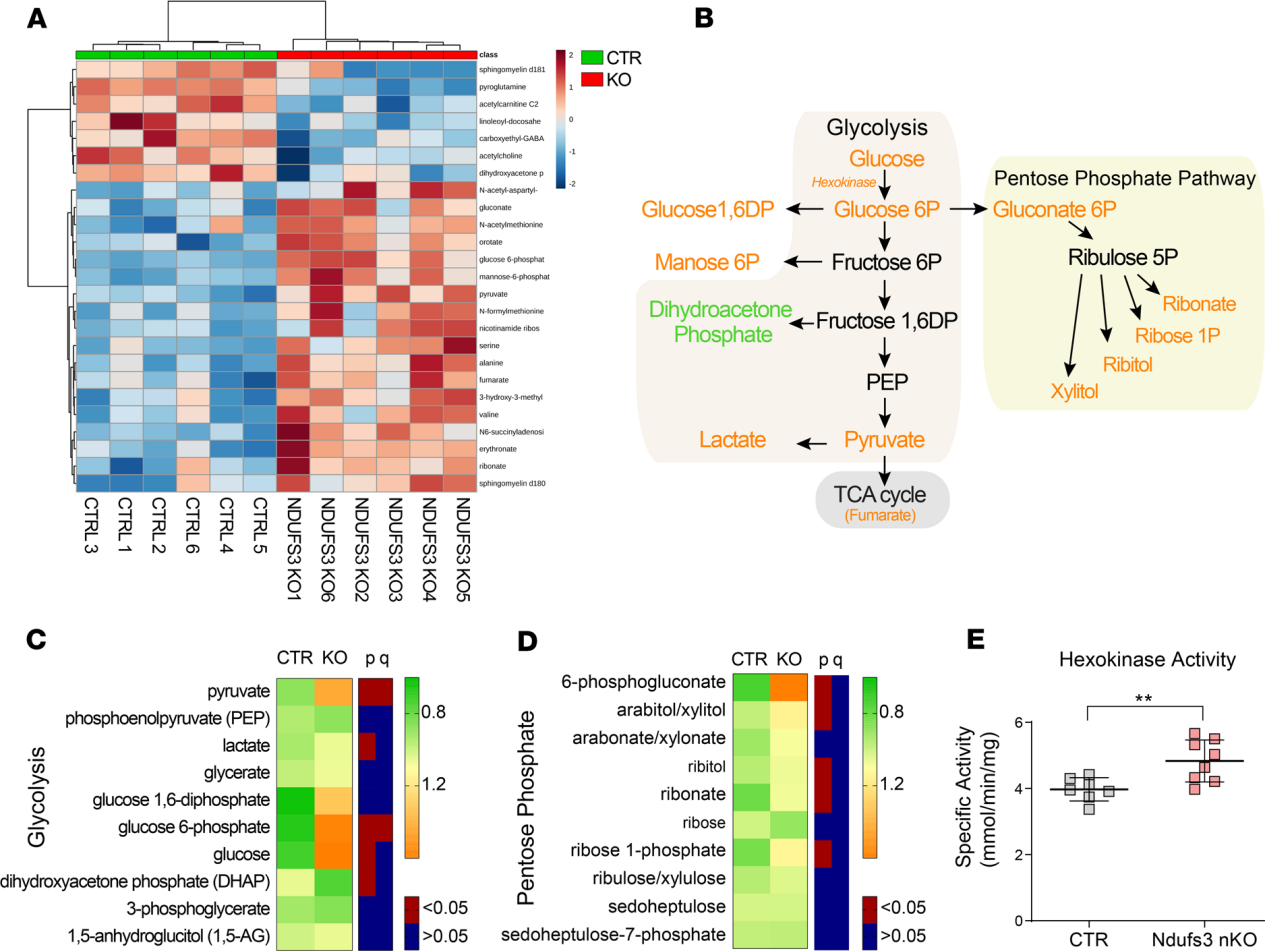
Glycolysis and pentose phosphate pathway metabolites changed in *Ndufs3*-KO brains. Cortices from 2.5-month-old *Ndufs3* nKO male mice, compared with those from control littermates, were used for the analysis (*n* = 6/group). (**A**) Euclidean Heatmap of top 25 metabolite changes showing a clear clustering of KO and controls. (**B**) Schematics of glycolysis and pentose phosphate pathways, with increased metabolites in orange. (**C**) Heatmap of average KO and control values for glycolysis intermediates. Both *P* and *q* values are shown. (**D**) Heatmap of average KO and control values for pentose phosphate pathway intermediates. Both *P* and *q* values are shown. (**E**) Hexokinase enzyme activity in brain homogenates of 4-month-old male *Ndufs3* nKO and control mice (*n* = 7–8/group). Groups were compared using Welch’s 2 sample *t* test, and *q* values were determined from the significant hits with *P* < 0.05. **P < 0.01.

*Ndufs3* nKO mice showed decreased CI activity in cortex homogenates starting at 2 months of age (Figure 2K). Therefore, we anticipated that alterations in OXPHOS function might impact energy metabolism in the brain at early ages. To study which putative metabolic adaptations were taking place early in the disease, we performed a global metabolomic analysis on cortex tissue from 2.5-month-old mice. Alterations in metabolites clearly clustered *Ndufs3* nKO and control brains (Figure 3A). Among the top hits, we found several glycolysis intermediates elevated in the *Ndufs3* nKO mice relative to controls (Figure 3, A–C). This result is compatible with a compensatory mechanism to counteract the possible decrease in ATP production. Interestingly, *Ndufs3* nKO brains did not show increases in the intermediate glycolysis metabolites, dihydroxyacetone phosphate, 3-phosphoglycerate, and phosphoenolpyruvate, but pyruvate and lactate were highly elevated (Figure 3B). This may indicate that increased levels of fructose 1,6-bisphosphate in *Ndufs3* nKO brain activated pyruvate kinase, which converts phosphoenolpyruvate to pyruvate, as well as poor utilization of Acetyl-CoA in the Krebs cycle. To complement the metabolomics study, we measured spectrophotometrically the specific activity of hexokinase, the enzyme that phosphorylates glucose to glucose-6-phosphate in cortex homogenates of 4-months-old mice. In agreement, hexokinase activity levels were increased in the cortices of 4-month-old *Ndufs3* nKO mice, indicating that this metabolic adaptation is sustained over time (Figure 3E). Lactate concentration in sera from 4-month-old *Ndufs3* nKO mice were not increased (Supplemental Figure 4D).

In addition to glycolysis, glucose is used in the pentose phosphate pathway (PPP) to support nucleotide synthesis and to provide NADPH (Figure 3D). Several PPP intermediates, such as 6-phosphogluconate, ribose-1-phosphate, ribitol, ribonate, and xylitol, were elevated in the *Ndufs3* nKO brains (Figure 3D). The increase of these metabolites in *Ndufs3* nKO brains may be connected to the increased glucose and glucose-6-phosphate levels, which increased the PPP initiator gluconate 6P (Figure 3, B and D).

Surprisingly, we have not found major changes in the TCA cycle metabolites, but there was an increase in levels of fumarate, the product of the SDH or CII (Figure 3A). This result would suggest increased SDH activity in cortices of 2.5-month-old *Ndufs3* nKO mice. In addition, *Ndufs3* nKO brains presented variations in other metabolic pathways, such as the methionine, mevalonate, and sphingolipid metabolism pathways (Supplemental Figure 4). Altogether, these results indicated a metabolic adaptation in the *Ndufs3* nKO mice that favored glucose utilization.

### Lack of NDUFS3 is associated with neuroinflammation in cortex and hippocampus and neuronal death in hippocampus.

**Figure 4:**
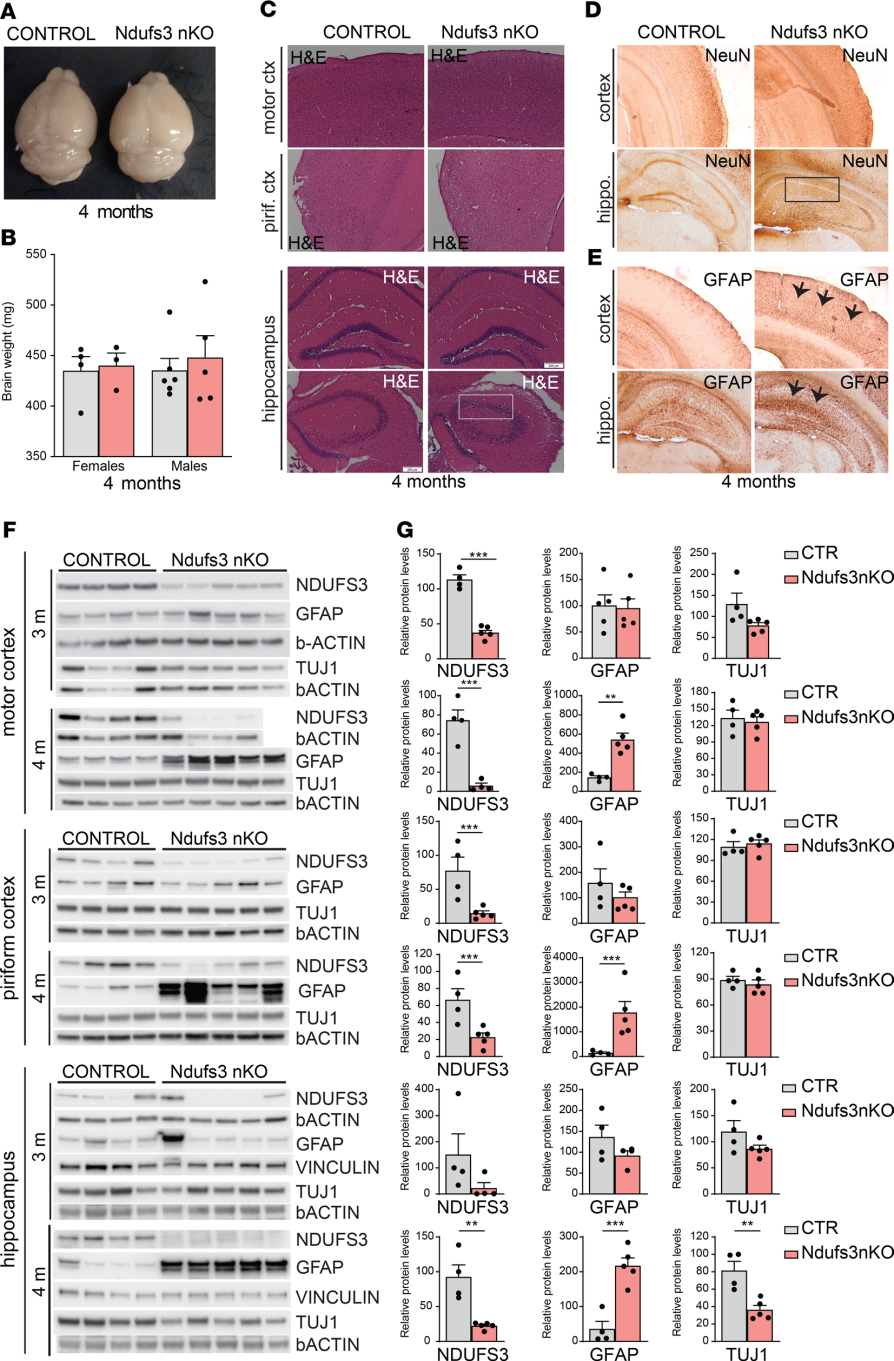
Lack of NDUFS3 in neurons leads to general neuroinflammation and neuronal cell loss in hippocampus. (**A**) Gross brain morphology of 4-month-old *Ndufs3* nKO mice revealed no changes. (**B**) Brain weight of 4-month-old *Ndufs3* nKO and control (CTR) female and male mice (*n* = 4–6). (**C**) H&E staining of cortical regions (first and second rows) of 4-month-old animals showing no apparent difference in morphology between control and *Ndufs3* nKO mice. H&E staining of hippocampal regions (third and fourth rows) of *Ndufs3* nKO mice showing less nuclei staining in the CA3 pyramidal layer (framed by a white rectangle). Original magnification, ×10. (**D**) Immunohistochemical images of NeuN staining on cortex (first row) and hippocampus (second row) of 4-month-old animals showing fewer neurons in hippocampi of *Ndufs3* nKO mice in the CA1 pyramidal layer (framed by a black rectangle). Original magnification, ×10. (**E**) Immunohistochemical images of GFAP staining on different brain regions of 4-month-old animals showing increased inflammation in the cortex and hippocampus regions of *Ndufs3* nKO mice (arrows). (**F**) Western blots and (**G**) relative quantification of protein homogenates from motor cortices, piriform cortices, and hippocampi of control and *Ndufs3* nKO animals at 3 and 4 months of age, probing for neuronal marker TUJ1, astrocyte activation (GFAP), and NDUFS3. β-Actin and vinculin antibodies were used as loading controls. GFAP levels were increased in all regions of nKO mice at 4 months of age. TUJ1 levels were decreased at 4 months of age in hippocampi of nKO mice. Data are represented as mean ± SEM (*n* = 4–5/group). *P* values were determined by Student’s *t* test. **P < 0.01, ***P < 0.001

In order to understand the basis for the phenotype, we first analyzed the gross anatomy of the brains. Brain isolated from 4-month-old *Ndufs3* nKO male and female mice did not show significant differences in weight when compared with controls (Figure 4, A and B). H&E staining of cortical regions from 4-month-old animals did not show obvious anatomical alterations (Figure 4C). However, hippocampal regions showed reduced nuclei staining (Figure 4C, inset).

We performed immunohistochemical analysis with anti-NeuN as neuronal marker on brains from 4-month-old animals (Figure 4D) and Western blots with anti-TUJ1 antibody on homogenates from different brain regions (motor cortex, piriform cortex, and hippocampus) of 3- and 4-month-old *Ndufs3* nKO animals (Figure 4, F and G). We did not detect changes in the neuronal population in the motor or piriform cortex. However, we detected reduced NeuN staining in the hippocampus, suggesting neuronal cell death at 4 months of age (Figure 4D, inset). This result was corroborated by Western blot analysis. Homogenates from 4-month-old *Ndufs3* nKO mice showed a 60% reduction in TUJ1 levels, indicating that neuronal NDUFS3 depletion caused neuronal cell death in the hippocampus (Figure 4, F and G). No differences in TUJ1 levels were detected in motor and piriform cortices of 4-month-old nKO mice (Figure 4, F and G).

Because activation of astrocytes and microglia is a common feature of neuropathology, we analyzed the extent of gliosis in these animals. We performed immunohistochemistry and Western blot analysis with an antibody against glial acidic fibrillary protein (GFAP) on animals at different ages and from different brain regions (motor cortex, piriform cortex, and hippocampus). GFAP levels in the *Ndufs3* nKO were similar to control at 3 months, but they were markedly increased at 4 months of age in motor cortex, piriform cortex and hippocampus (Figure 4E, arrows, and Figure 4, F and G). To analyze if the lack of NDUFS3 induced microglial activation, we performed Western blot analysis with the antibody against the ionized calcium-binding molecule 1 (IBA-1). However, we did not detect increased levels of IBA-1 protein in homogenates from either the motor cortices or the hippocampi of *Ndufs3* nKO mice (see below)**.**

Altogether, our results show that the ablation of NDUFS3 in CaMKIIa-expressing neurons induced a mitochondrial encephalopathy characterized by massive astrocyte activation in all brain regions analyzed and neuronal cell death localized to the hippocampus at 4 months of age.

### Metformin treatment delayed the onset of the disease in Ndufs3 nKO mice, particularly in female mice.

**Figure 5:**
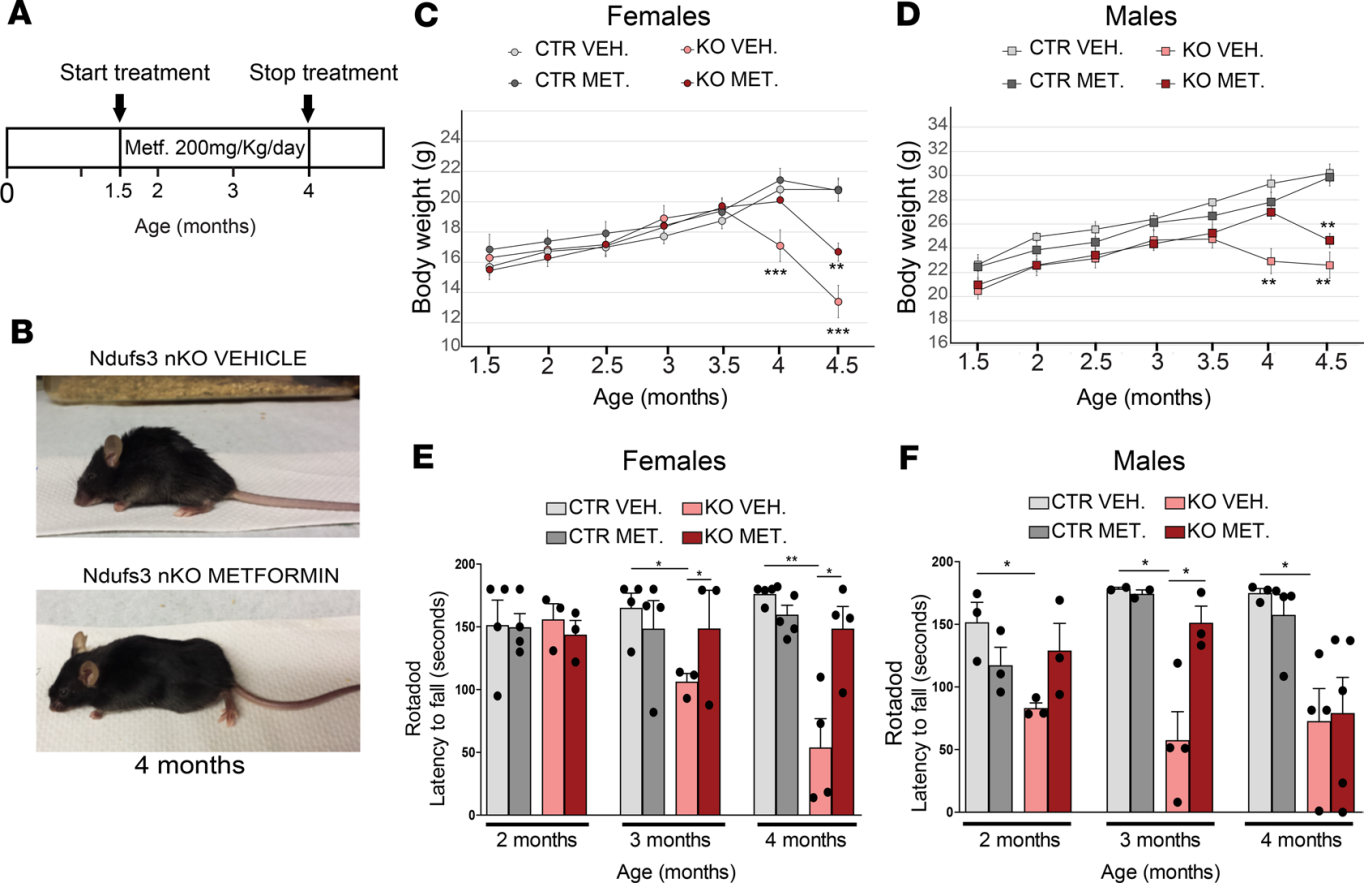
Metformin treatment delays the onset of the phenotype in *Ndufs3* nKO mice. (**A**) Schematic representation of the protocol used for metformin treatment. (**B**) Representative images of 4-month-old vehicle-treated *Ndufs3* nKO female mice and metformin-treated *Ndfus3* nKO female mice. (**C**) Body weight comparison over time of female mice: vehicle-treated *Ndufs3* nKO (pink circles; *n* = 4); metformin-treated *Ndufs3* nKO (red circles; *n* = 5), vehicle-treated controls (light gray circles, *n* = 6), and metformin-treated controls (dark gray circles, *n* = 3). (**D**) Body weight comparison over time of male mice: vehicle-treated *Ndufs3* nKO (pink squares; *n* = 6), metformin-treated *Ndufs3* nKO (red squares; *n* = 3), vehicle-treated controls (light gray squares, *n* = 7), and metformin-treated controls (dark gray squares, *n* = 5). (**E** and **F**) Rotarod performance of control and *Ndufs3* nKO female mice (**E**) and male mice (**F**) of 2, 3, and 4 months of age (*n* = 3–5/group). Statistical significance was determined using 1-way ANOVA. Pairwise Bonferroni’s post tests were used to compare different groups in all panels. Data are presented as mean ± SEM. *P < 0.05, **P < 0.01, ***P < 0.001.

**Figure 6:**
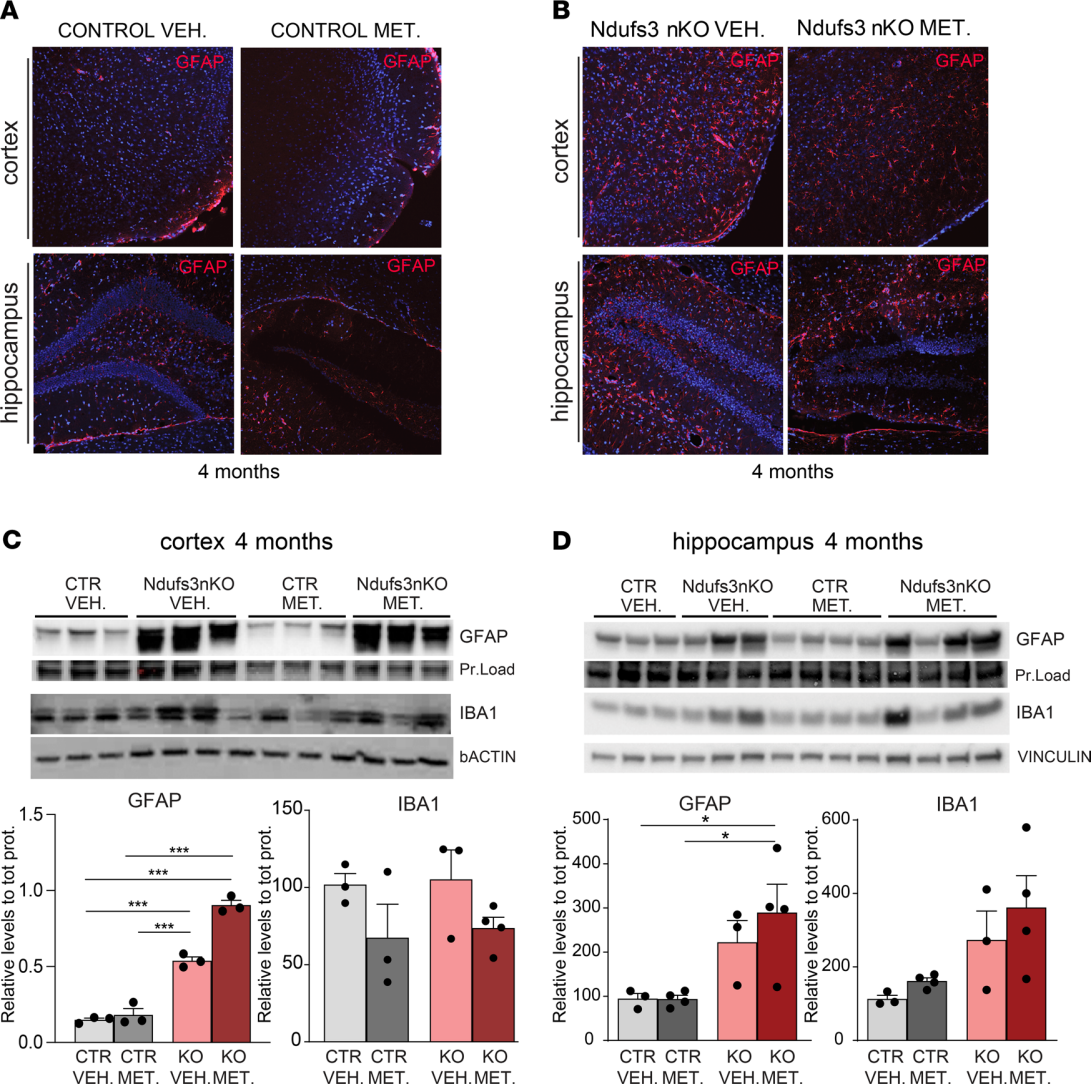
Metformin has no significant effect on GFAP activation in cortices from 4-month-old *Ndufs3* nKO mice. (**A** and **B**) Immunohistochemical images of GFAP on cortex and hippocampus regions of 4-month-old controls (**A**) and *Ndufs3* nKO (**B**) animals treated with vehicle versus metformin. Images in **B** show increased inflammation in the cortex and hippocampus (red signal) of *Ndufs3* nKO mice compared with control (CTR) mice. (**C**) Western blots and relative quantification of protein homogenates from motor cortices of control and *Ndufs3* nKO mice at 4 months of age, probing for astrocytes (GFAP) and microglia (IBA1). Quantification was normalized for protein loading or β-actin. (**D**) Western blots and relative quantification of protein homogenates from hippocampi of control and *Ndufs3* nKO mice at 4 months of age, probing for astrocytes (GFAP) and microglia (IBA1). Quantification was normalized for protein loading or vinculin. Data are represented as mean ± SEM (*n* = 3–4/group). *P* values were determined by ANOVA followed by Bonferroni’s post hoc comparison. *P < 0.05, ***P < 0.001.

**Figure 7:**
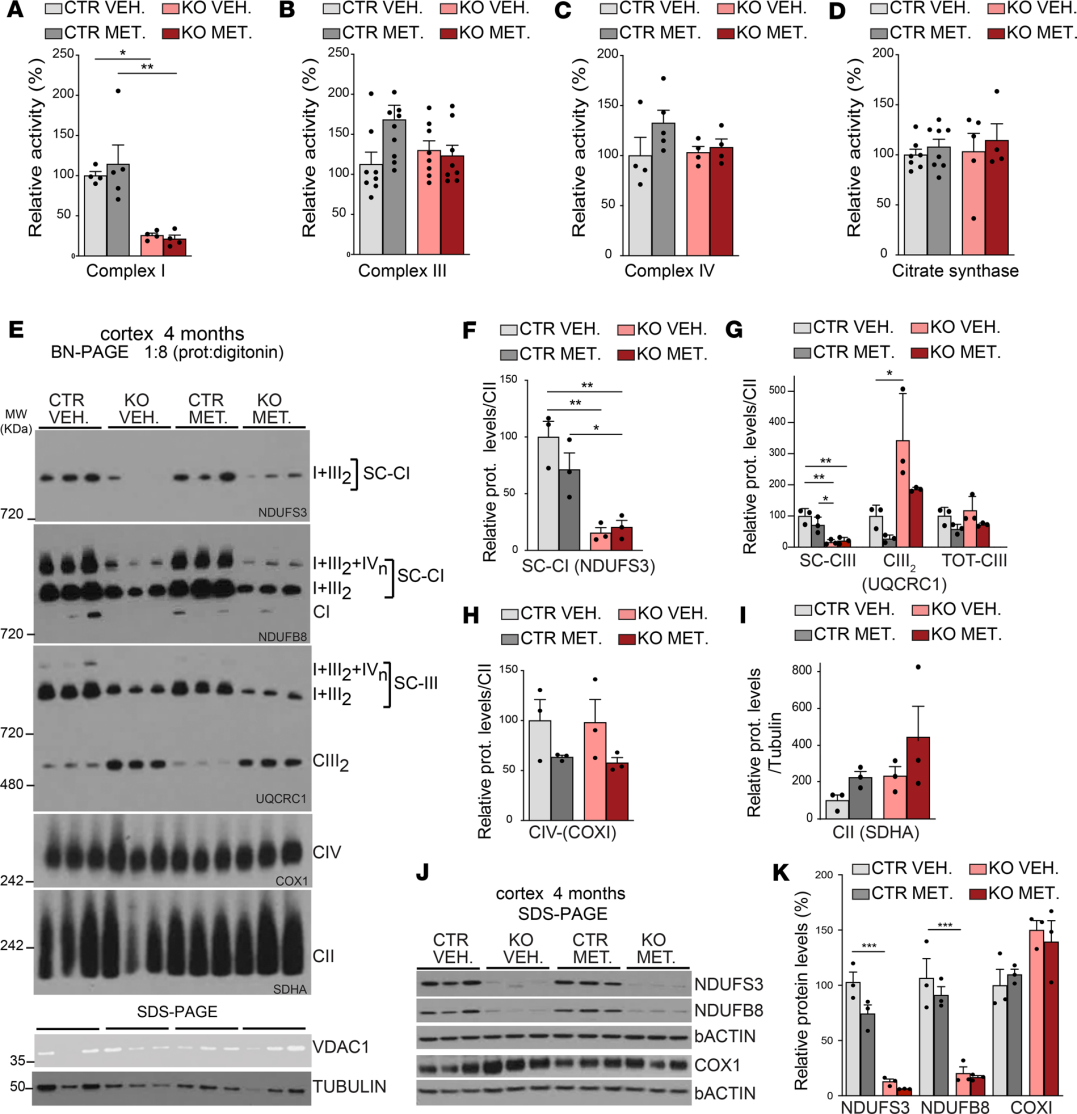
Metformin treatment does not affect OXPHOS complex activities or OXPHOS steady-state levels in *Ndufs3* nKO mice. (**A**–**D**) Spectrophotometric complex I/citrate synthase, complex III/citrate synthase, and complex IV/citrate synthase activity ratios and citrate synthase /protein activity ratios were measured in cortex homogenates from 4-month-old female mice. Data are represented as mean ± SEM (*n* = 4–9/group). *P* values were determined by 1-way ANOVA followed by Bonferroni’s post hoc comparison. (**E**) Steady-state levels of supercomplexes of complex I, III, and complexes IV and II measured by BN-PAGE in cortex homogenates from 4-month-old female mice using antibodies against NDUFS3 and NDUFB8 (complex I subunits), UQCRC1 (complex III), COX1 (complex IV), and SDHA (complex II) subunits. (**F**–**I**) Quantification of the BN-PAGE showed in **E** (*n* = 3/group). (**J** and **K**) Western blot and quantification of protein homogenates from motor cortices of 4-month-old female mice treated with vehicle or metformin using antibodies NDUFS3 and NDUFB8 (complex I subunits), COX1 (complex IV subunit), and β-actin. Data are represented as mean ± SEM (*n* = 3/group). *P* values were determined by 1-way ANOVA followed by Bonferroni’s post hoc comparison. *P < 0.05, **P < 0.01, ***P < 0.001.

Metformin increases life span and health span in control mice by shifting the global gene expression in a fashion that resembles the changes induced by caloric restriction (27). Moreover, metformin crosses the blood-brain barrier as previously reported (34). The OXPHOS defect and the metabolic adaptations toward increased glycolysis suggested that boosting glucose uptake in neurons could be beneficial. Metformin is known to increase glucose uptake in several tissues, including the CNS (35). In order to analyze the effect of chronic metformin treatment in this mouse model, we administered the drug by intraperitoneal injection (200 mg/kg/d), starting at 1.5 months of age, when the mice were asymptomatic (Figure 5A). This dose has been proved to be active in mice and to promote neurogenesis when administered from P1 (36). Daily metformin treatment improved the physical appearance of the *Ndufs3* nKO mice at 4 months of age (Figure 5B) and delayed by 2 weeks the weight loss in both male and female *Ndufs3* nKO mice (Figure 5, C and D). Performance in the rotarod assay was determined at 2, 3, and 4 months of age. Metformin delayed the onset of the motor phenotype in both male and female *Ndufs3* nKO mice (Figure 5, E and F). The effect was more pronounced in female mice, which performed as the control group at 4 months of age (Figure 5E). By contrast metformin-treated *Ndufs3* nKO male mice showed recovered motor coordination at 2 and 3 months but not at 4 months of age (Figure 5F). Metformin-treated *Ndufs3* nKO mice also showed a greatly attenuated clasping phenotype; it was present in 100% of 4-month-old *Ndufs3* nKO mice (Supplemental Figure 5B). When we tested the locomotor nocturnal activity, vehicle-treated *Ndufs3* nKO female mice showed increased activity, whereas metformin-treated *Ndufs3* nKO male mice did not (Supplemental Figure 5C). Moreover, metformin-treated *Ndufs3* nKO female mice did not show ataxia and behaved similar to control mice when handled by the investigator (Supplemental Video 2). Despite the phenotypic improvements, metformin treatment did not extend life span in *Ndufs3* nKO male mice (Supplemental Figure 5D). We sacrificed all female mice treated with metformin and vehicle to perform enzymatic assays, Western blots, BN-PAGE, and immunohistochemistry shown in Figures 5–7. Therefore, we did not determine if metformin affects survival in female mice. Still, our data showed that metformin treatment delayed the onset of the phenotype in *Ndufs3* nKO mice and had a stronger effect in female mice than in male mice.

### Metformin does not reduce neuronal death in the CNS of Ndufs3 nKO mice.

Metformin has been reported to have potent antiinflammatory properties in mice and humans (37, 38) and to prevent neurodegeneration in mammalian animal models of Alzheimer’s disease (28), multiple sclerosis (29), and Huntington’s disease (30). To understand if metformin’s effect on the motor phenotype of *Ndufs3* nKO mice was due to reduced neuroinflammation, we analyzed glial activation in 4-month-old female mice. By immunohistochemistry we observed an apparent mild reduction of GFAP^+^ cells in cortices of metformin-treated *Ndufs3* nKO female mice compared with that in vehicle-treated *Ndufs3* nKO mice (Figure 6, A and B). However, GFAP Western blots in cortex homogenates of 4-month-old mice did not show significant differences (Figure 6C). Accordingly, metformin treatment did not alter GFAP levels in hippocampus homogenates (Figure 6, B and D). We also measured levels of IBA1 (marker of microglia) without detecting any change (Figure 6, C and D). The levels of inflammatory markers, including Casp1, cleaved Casp1, and NRLP3, were not improved by metformin and, in the case of IRF3, were modestly increased (Supplemental Figure 6A).

Since *Ndufs3* nKO mice showed neuronal cell death in the hippocampus, we also analyzed the effect of metformin on hippocampal neurodegeneration by measuring TUJ1 content (Supplemental Figure 6B). No differences were found in the levels of TUJ1 between groups, suggesting that metformin did not counteract the neuronal cell death observed in the hippocampus region of 4-month-old *Ndufs3* nKO mice.

### Chronic metformin treatment does not alter OXPHOS function in Ndufs3 nKO mice.

It has been reported that metformin can inhibit CI activity (39). To determine a possible effect of metformin in OXPHOS activity, we analyzed homogenates from cortices of 4-month-old animals. Rotenone-sensitive CI activity of *Ndufs3* nKO mice was dramatically decreased to approximately 30% of control levels (Figure 7A). Metformin did not affect rotenone-sensitive CI activity in WT controls or in *Ndufs3* nKO mice (Figure 7A). Activities of respiratory CIII, CIV, and citrate synthase of *Ndufs3* nKO mice were comparable to those of control samples and were not altered by metformin treatment (Figure 7, B–D). Next, to determine the abundance of CI in supercomplexes, cortex homogenates of 4-month-old mice were treated with a mild detergent (protein/digitonin ratio = 1:8) and analyzed by BN-PAGE. The decrease in the levels of the supercomplexes containing CI subunits (NDUFS3 and NDUFB8) in the *Ndufs3* nKO mice was not recovered by metformin treatment (Figure 7, E and F).

The total levels of CIII were not changed in the cortices from *Ndufs3* nKO mice, as shown in the membrane probed for UQCRC1, but the distribution was altered (Figure 7, E and G). In control animals, CIII was predominantly present in supercomplexes, just as for CI (Figure 7E), whereas in *Ndufs3* nKO mice CIII was more abundant as free complex (Figure 7, E and G). This result may be explained by the reduction in CI levels seen in *Ndufs3* nKO mice (Figure 7, E and F). Metformin treatment did not affect CIII levels or distribution. CIV and CII levels were determined using antibodies against the mtDNA-encoded subunit COX1 and the nDNA-encoded subunit SDHA, respectively. No differences were found between groups (Figure 7, E, H, and I). Although there was a trend toward higher CII levels in the cortices of the metformin-treated groups (both control and *Ndufs3* nKO), it did not reach statistical significance (Figure 7I).

In order to determine a possible effect of metformin in the NDUFS3 protein levels, we performed Western blot analysis in cortex homogenates. We did not detect increased levels of NDUFS3 in mice treated with metformin (Figure 7, J and K). We have also found no differences in NDUFB8 between metformin and vehicle-treated mice (Figure 7, J and K). As observed before (Figure 2), the CIV subunit COX1 was increased in *Ndufs3* nKO cortices compared with those of control mice (Figure 7, J and K). No differences were found in the COX1 levels between metformin and vehicle-treated *Ndufs3* nKO groups. Altogether, these results indicated that the chronic metformin treatment did not restore CI function in the cortex. In addition, we did not detect changes in lactic acid levels in serum or in hippocampus homogenates (Supplemental Figure 6, C and D).

### Metformin did not alter the metabolic status of the AMPK and mTOR pathways in the Ndufs3 nKO mice.

The mechanism of action of metformin involves several pathways and molecular interactions. A major effect of the drug is to enhance the phosphorylation of AMP-activated protein kinase (AMPK) (27, 40). Therefore, to determine if metformin activates AMPK in brain tissue in our mice, we measured the steady-state levels of phosphorylated and total AMPK and one of its main targets, acetyl-CoA carboxylase (ACC; we measured both phosphorylated ACC and total ACC), in homogenates from the cortex and hippocampus. We did not observe a significant increase of the phospho-AMPK versus AMPK total levels in tissues of the metformin-treated groups (Supplemental Figure 7, A–D).

Metformin is also known to inhibit mTOR signaling in vitro (41) and in vivo (42), mostly through AMPK. Steady-state levels of phosphorylated and total mTOR and its downstream target, ribosomal protein S6 (RSP6) kinase, in homogenates from the cortex and hippocampus showed only a trend toward higher levels of phosphorylated RSP6 in the homogenates of metformin-treated *Ndufs3* nKO female mice (Supplemental Figure 7, E–G). We also measured the levels of eukaryotic translation initiation factor 4E–binding (eIF4E-binding) protein 1 (4E-BP1), a well-known substrate of the mTOR signaling pathway. Increased levels of phosphorylated 4E-BP1 versus total 4E-BP1 were detected in cortex homogenates of *Ndufs3* nKO female mice, indicating a possible inhibition of the mTOR pathway (Supplemental Figure 7, E and G). A trend toward an increase was also observed in hippocampi of *Ndufs3* nKO female mice treated with metformin; however, it did not reach the statistical significance (Supplemental Figure 7, F and H). In summary, although the metabolic regulators AMPK and mTOR could play a role in the metformin-induced improved phenotype, we found only small evidence that they do.

## Discussion

Prognosis for mitochondrial encephalopathies associated with CI dysfunction is very poor, and there is no cure. Therefore, the creation of animal models is extremely important to understand the mechanisms of neurodegeneration and to develop new therapies. We created a potentially novel mouse model of neuronal CI deficiency by specifically deleting the *Ndufs3* gene in forebrain neurons. Two patients diagnosed with Leigh syndrome have been reported to date to carry *Ndufs3* mutations, both with CNS involvement (21, 23).

For mitochondria of nonsynaptic origin, CI activity needs to be decreased to approximately 40% before major changes in rates of oxygen consumption and ATP synthesis are observed, suggesting that they have larger reserves of activity and must be inhibited to a large extent before OXPHOS is severely compromised (43). This would explain why in our mouse model we detected impaired balance and impaired motor coordination when CI activity reduction in cortex is partial (~67% of control levels), while we observed neurodegeneration and gliosis only later, when CI activity reaches a drastic reduction to less than approximately 25% of the control levels.

Cortex metabolomic analysis of 2.5-month-old mice showed an abnormal metabolic profile in the *Ndufs3* nKO mice, which included accumulation of pyruvate, lactate, and glycolytic intermediates, consistent with clinical reports of Leigh syndrome (44, 45). Likewise, a similar increase in glycolytic intermediates was detected in the whole-brain metabolomics of the *Ndufs4*^–/–^ mice (26). Under normal circumstances, pyruvate is converted to acetyl-CoA by the pyruvate dehydrogenase complex, with the concomitant reduction of NAD^+^. The decreased NAD^+^/NADH ratio caused by an OXPHOS defect inhibited the conversion of pyruvate to acetyl-CoA, resulting in increased pyruvate levels (Figure 3A). Increased pyruvate levels can also derive from increased glycolytic activity in an attempt to compensate for decreased mitochondrial ATP production or perhaps as a result of decreased conversion of pyruvate to acetyl-CoA to support the TCA cycle. Increased glucose uptake might also lead to increased glycolysis.

Our results suggest that cortical neurons metabolically adjust to some extent to counteract a partial CI deficiency. These neuronal metabolic adaptations allowed *Ndufs3* nKO mice to grow at the same rate as the controls and to display similar nocturnal ambulatory activity and rodent stereotyped behavior for the first 3 months of their life. Only the execution of more complex tasks, such as rotarod, where coordination and equilibrium are required, were impaired. One of the adaptive mechanisms observed in mice with mitochondrial defects is an increase of mitochondrial mass and biogenesis. We have recently described the *Ndufs3* smKO model, a conditional KO mouse lacking the NDUFS3 subunit exclusively in skeletal muscle (46) that develops a progressive myopathy and has a reduced life span. Already, at 2 months of age, these mice showed increased levels of PGC-1α (a transcriptional coactivator regulating mitochondrial biogenesis), COXI (CIV subunit), and SDHA (CII subunit), and at 4 months of age VDAC-1 levels were doubled. These results indicated that the absence of NDUFS3 in skeletal muscle induced a robust mitochondrial proliferation as a compensatory mechanism. However, the CNS does not appear to have the same metabolic flexibility as skeletal muscle. In the *Ndufs3* nKO model, although mtDNA levels were increased 1.7-fold and the levels of COXI and SDHA were increased from 3 months, the levels of VDAC1 and TIM23 were similar to those of controls in both the cortex and hippocampus. Likewise, citrate synthase activity (enzyme of the TCA cycle, whose levels usually correlate with mitochondrial content) in cortex was comparable to that in controls. We had similar observations in the brains of *Ndufa5* nKO mice (47). These observations suggest that the isolated CI deficiency in brain induces increased expression of a subset of mitochondrial proteins, rather than a general induction of mitochondrial biogenesis to compensate for the energetic impairment. These findings highlight a fundamental difference between muscle and CNS in terms of metabolic adaptations and mitochondrial levels flexibility.

Because our mouse model recapitulates many of the phenotypes of CI deficiency in humans and had an altered glucose-related metabolism, we decided to test the effect of metformin on the progression and development of the disease and found that metformin delayed the onset and attenuated the progression of the disease in *Ndufs3* nKO mice. Some reports have pointed out that CI inhibition is involved in the mechanism of action for metformin in vitro (48–50) and in vivo in skeletal muscle from healthy and diabetics rats (39). Our data clearly show that chronic treatment with therapeutic doses of metformin did not affect the residual CI activity in the cortices of *Ndufs3* nKO mice. Similarly, metformin did not alter mitochondrial function or CI in control animals. Moreover, and in agreement with our results, other reports have shown that metformin does not affect mitochondrial respiratory capacity in vivo (51) or CI activity in patients with type 2 diabetes (52). The in vivo relevance of metformin’s in vitro inhibition of CI is still under debate (53).

Metformin-associated lactic acidosis (MALA) has been recognized for several decades (54). However, the risk of MALA is still debated, and large clinical trials did not find it to cause lactic acidosis in patients with diabetes (55) or traumatic brain injury (56). In the cases reported, underlying comorbidities, such as renal failure or hypoxia could be the underlying cause (57). A CI deficiency in the CNS could be consider a risk factor, and, in fact, metformin is not recommended for patients with mitochondrial diseases (58). In our neuronal CI-deficient mouse model, metformin did not alter lactate levels in either hippocampus homogenates or in the serum. Because the CI defect was restricted to neurons, systemic lactic acidosis may not be an issue in this model. However, care should be exercised when dealing with patients with underlying lactic acidosis.

At the physiological level, metformin has been reported to facilitate glucose utilization (59); activate mitochondrial biogenesis in endothelial cells (60, 61); reduce mitochondrial oxidative stress in animals (62); and decrease inflammation (63). Our metabolome results showed that the *Ndufs3* nKO brain has adapted to increase glycolysis and glucose-related metabolism. Peripheral uptake of [^3^H]-2-DG, an analog of glucose, was shown to increase significantly, mostly in the brain and small intestine, as an acute response to oral metformin in fasted mice (64). Therefore, metformin would enhance glucose availability to the CNS of *Ndufs3* nKO mice, which would improve synaptic function. In addition, metformin can have several auxiliary beneficial effects, such as decreasing inflammation; however, even if present in our model, metformin did not show improvements. Metformin has been shown to activate the AMPK pathway. In our experiments, we found no significant changes in AMPK pathway activation. Likewise, the analyses of the mTOR pathway did not show major changes, with exception of increased phosphorylation of 4E-BP1. Rapamycin-induced inhibition of mTOR was shown to improve survival and health in the *Ndufs4^−/−^* mouse model of Leigh syndrome (26), so even small effects on mTOR inhibition could be beneficial in this context. However, we have not been able to show significant changes in any of these processes, suggesting that, despite better performance, as reflected in improved behaviors, defective neurons are still dying at approximately the same rate due to the CI defect. Therefore, the phenotypic improvement may reflect a temporary enhanced synaptic function, which delays onset but is not able to prevent the neuronal loss due to the lack of CI.

In summary, we created and characterized a potentially novel model of the CI defect in the CNS and showed that it upregulated glucose metabolism in brain. We also showed that metformin promoted modest neurological improvement and attenuation of the disease phenotype. Although this compensatory mechanism did not change neuronal fate, the fact that it did not exacerbate the CI defect is noteworthy. These observations warrant further investigation in metformin use in patients with mitochondrial diseases without lactic acidosis. The potential for exacerbating lactic acidosis was not observed in this model, but it should be carefully monitored if used in the clinical practice.

## Methods

### Creation of Ndufs3 nKO mice.

We acquired the transgenic mouse for the floxed *Ndufs3* mice from the Knockout Mouse Project repository (MMRRC:059252-UCD, Supplemental Figure 1). The male mouse purchased was breed with female C57BL/6J mice to establish the colony. The transgenic mice heterozygous for the *Ndufs3*-floxed allele were subsequently crossed between them to obtain homozygous floxed mice (*Ndufs3*^fl/fl^ mic). To generate the *Ndufs3* neuron-specific KO mice, *Ndufs3*^fl/fl^ were mated with the *CaMKIIa*-Cre transgenic mice supplied by Scott Zeitlin (University of Virginia, Charlottesville, Virginia, USA). Animals for this study were obtained following the scheme described in Supplemental Figure 1C. Controls and conditional KO mice were obtained from the same litters. The presence of the WT, floxed *Ndufs3* genes, and the Cre transgene was detected by PCR using the following pairs of primers: *Ndufs3* gene, forward F1, 5′-GCAAAGATTGCCCAACACAG-3′, and reverse R1, 5′-CTCCAAGCCCTCCCTGAAG-3′ and R2, 5′-TGCCCTTTCTCACCTTTAGTCC-3′. The PCR product obtained from the WT *Ndufs3* gene with primers F1 and R1 was 333 bp in length, and the PCR product from the floxed *Ndufs3* gene with primers F1 and R1 was 473 bp. The PCR product from the floxed *Ndufs3* gene with primers F1 and R2 was 1692 bp in length. After deletion, PCR product from the floxed *NdufS3* gene with primers F1 and R2 was 408 bp. The gene ID for *NdufS3* is 68349. For *CaMKIIa-Cre* transgene, the forward primer used was 5′-GCGGTCTGGCAGTAAAAACTATC-3′ and the reverse was 5′-GTGAAACAGCATTGCTGTCACTT-3′ (287 bp). A PCR product of 409 bp was amplified as an internal positive control of the PCR reaction using the following primers obtained from The Jackson Laboratory: oIMR7338 F, 5′-CTAGGCCACAGAATTGAAAGATCT-3′ and oIMR7339R, 5′-GTAGGTGGAAATTCTAGCATCATCC-3′.

All animals used in this work had a C57BL/6J background and were backcrossed for at least 10 generations. Mice were housed in a virus antigen–free facility at the University of Miami, Division of Veterinary Resources, in a 12-hour-light/dark cycle at room temperature and fed ad libitum.

### Ambulatory nocturnal activity behavioral test.

Spontaneous ambulatory movement of mice was recorded using the Opto-M3 activity meter (Columbus Instruments) equipped with infrared beams along the cage. Movement was counted as the number of times the infrared beams were disrupted. Mice were housed individually in a new cage 30 minutes before their daily dark cycle, and ambulatory counts were recorded for a period of 12 hours (6:30 pm to 6:30 am).

### Open-field behavioral test.

Open field (Med Associates Inc.) is a sensitive method for measuring gross and fine locomotor activity. It consists of a chamber and a system of 16 infrared transmitters that record the position of the animal in the 3D space. This system can record not only the horizontal movement, but also the rearing activity. For our study, the animals were placed in the chamber 30 minutes before the test, and the locomotor activities were recorded for 30 minutes.

### Rotarod behavioral test.

Mouse motor coordination was tested at different ages using a Rotarod (IITC 755 Life Sciences) set at a ramp speed of 6–20 rpm over 180 seconds. The test consisted of 3 trials performed for each animal at the corresponding age, and the latency to fall was recorded. Mice that completed the task received a final latency time of 180 seconds. Animals were trained in the rotarod twice per trial, over 3 trials, each about 2 weeks before the first test.

### Metformin treatment.

Mice were treated with metformin (200 mg/kg, Toronto Research Chemical Inc., M258815), which was dissolved in saline and administrated via intraperitoneal injection daily starting at 1.5 months of age.

### Western blots.

Protein extracts were prepared from the cortex and hippocampus regions and homogenized in PBS containing a protease inhibitor mixture (Roche). Upon use, SDS was added to the homogenate at the final concentration of 4%. Homogenates were then centrifuged at 14,000*g* at 4°C, and the supernatant was collected for analysis. Protein concentration was determined by the Lowry assay using the BCA kit (Bio-Rad). Approximately 20–40 μg protein was separated by SDS-PAGE in 4%–20% acrylamide gels and transferred to PVDF membranes (Bio-Rad). Membranes were blocked with 5% nonfat milk in 0.1% Tween-20 in PBS and subsequently incubated with specific antibodies, which were incubated overnight at 4°C. Antibodies against NDUFS3, NDUFB8, NDUFA9, SDHA, SDHB, UQCRC1, UQCRC2, COXI, and VDAC1 were obtained from Abcam (1:1000); β-actin and tubulin were obtained from MilliporeSigma (1:5000); GFAP was obtained from Cell Signaling Technology (1:2000); IBA1 was obtained from Wako (1:500); and TUJ1 was obtained from Covance (1:10,0000). AMPKa, phospho-AMPKa (Thr172), acetyl-CoA carboxylase, phospho-acetyl-CoA carboxylase (Ser79), mTOR, phospho-mTOR (Ser2448), phospho-p70 S6 kinase (Thr389), phospho-p70 S6 kinase (Ser371), and phospho-4E-BP1 (Thr37/46) were obtained from Cell Signaling Technology and used at 1:1000. Secondary antibodies conjugated to horseradish peroxidase (Cell Signaling Technology) were used, and the reaction was developed by chemiluminescence using SuperSignal West reagent (Thermo Fisher Scientific). Blots were visualized with Chemidoc Imaging System (Bio-Rad). Optical density measurements were taken by software supplied by Bio-Rad.

### Enzymatic activity assays.

Cortex homogenates were prepared in PBS containing complete protease inhibitor cocktail (Roche diagnostics) in a volume 10 times the weight of the homogenates. The tissue was disrupted by 10–15 strokes using a motor-driven pestle. Homogenates were centrifuged at 1000*g* for 5 minutes, and supernatants were used for enzymatic assays. The activities of CI, CIII, CIV, and citrate synthase were measured spectrophotometrically as described previously (65, 66). Protein concentrations were determined using the Bio-Rad Bradford Assay Kit with BSA as standard. Specific activity was determined as μmoles/min/mg protein, and final values were represented as a percentage of the control values.

### BN-PAGE.

To identify and estimate the levels of respiratory supercomplexes, homogenates from cortices were treated with digitonin (ratio 1:8, protein/digitonin; Roche), and mitochondrial complexes were separated by BN–PAGE in 3%–12% acrylamide gradient gels (Invitrogen) (67, 68). To determine the levels of isolated respiratory complexes, cortex homogenates were treated with Dodecylmaltoside 1% final concentration (MilliporeSigma) and separated by BN-PAGE in 4%–16% acrylamide gradient gels (Invitrogen). 10 μg of protein was separated by PAGE, transferred to a PVDF membrane (Bio-Rad), and incubated sequentially with antibodies against several subunits of the different mitochondrial respiratory complexes. To detect the activity of CI in gel, mitochondrial complexes treated with Lauryl Maltoside (MilliporeSigma) and separated in 4%–16% gels were incubated with 14 mM NADH and 1 mg/mL nitroblue tetrazolium in Tris-HCL 0.1 M, pH 7.4, and incubated at 37°C for approximately 1 hour.

### Immunostaining.

Anesthetized mice were transcardially perfused with ice-cold PBS and 4% PFA. The brains were isolated, and regions of interest were dissected using a brain matrix, cryoprotected in sucrose 30%, and frozen in OCT. Frontal and sagittal sections were cut at a 20 μm thickness with a cryostat (Leica). Sections were blocked with 10% normal goat serum for 30 minutes at room temperature and then incubated with primary antibody anti-NeuN (1:1000, Chemicon) overnight at 4°C. Slides were then incubated with secondary antibody biotin-conjugated goat anti-mouse (KPL) for 1 hour at room temperature and Streptavidin-Peroxidase (KPL) for 30 minutes at room temperature. Staining was visualized using a solution of 0.05% 3,3′-diaminobenzidine, 50 mM Tris-HCl, pH 7.2, 0.02% H_2_O_2_. Images were captured with an optic microscope.

For immunofluorescence staining, sections were blocked with 10% normal goat serum for 1 hour at room temperature and permeabilized with 1% Triton X-100. Sections were incubated with primary antibody (GFAP, 1:500, Cell Signaling Technology; anti-NeuN, 1:1000, Chemicon) for 16 hours at 4°C. Slides were then incubated with Alexa Fluor secondary antibody for 1 hour at room temperature and mounted with Vectashield mounting medium (Vector Laboratories) for fluorescence. Images were captured with an Olympus BX51 confocal microscope.

### Quantitative PCR of genomic DNA.

Genomic DNA was extracted from cortex tissue using standard proteinase K, phenol, chloroform extraction, and isopropyl alcohol precipitation. The ratio of mtDNA to nDNA was determined by quantitative real-time PCR using 10 ng genomic DNA in a 20 μl reaction mixture using SsoFast EvaGreen Supermix (Bio-Rad), following PCR conditions stipulated by the manufacturer in a CFX96 Real-time PCR system (Bio-Rad). Primers for mtDNA were ND1-F, 5′-CAGCCTGACCCATAGCCATA-3′, and ND1-B, 5′-ATTCTCCTTCTGTCAGGTCGAA-3′, and those for genomic DNA were β-actin F, 5′-GCGCAAGTACTCTGTGTGGA-3′, and β-actin B, 5′-CATCGTACTCCTGCTTGCTG-3′. DNA amounts were quantified using the ddCt method and expressed as a ratio of ND1/β-actin.

### Plasma lactate measurements.

Blood was drawn from deeply anesthetized animals by cardiac puncture. Lactate levels were measured in plasma using Lactate Plus Lactate Meter (Nova Biomedical).

### Metabolomic analysis.

Cortex tissues from 2.5-month-old *Ndufs3* nKO male mice and male control littermates were dissected, deeply frozen, and analyzed by Metabolon (https://www.metabolon.com/). Six biological replicates were analyzed from each group. Further details related to the protocol are provided in the Supplemental Methods. Groups were compared using Welch’s 2-tailed *t* test, and *P* < 0.05 was considered statistical significant.

### Statistics.

GraphPad Prism 6 software was used for the presentation of the data. Two-tailed, unpaired Student’s *t* test was used to determine the statistical significance between 2 different groups. Multiple groups were compared using a 1-way ANOVA followed by Bonferroni’s post hoc comparison. Data are shown as the mean ± SEM. *P* values of less than 0.05 were considered significant.

### Study approval.

All experiments and animal husbandry were performed according to a protocol approved by the University of Miami Institutional Animal Care and Use Committee.

## Author contributions

SP designed the research, performed the experiments, analyzed and interpreted data, and wrote the manuscript. MP performed immunostainings and Western blots, analyzed and interpreted data, and contributed to the writing of the manuscript. SG helped with the mouse colony and dissected tissue. TA performed Western blots. FD assisted with the BN-PAGE analysis, performed experiments, and contributed intellectually to the research. CTM planned the project together with SP and contributed to the writing of the manuscript. All authors edited the manuscript.

## Supplementary Material

supplemental data

supplemental Video 1

supplemental Video 2

## Version 1 11/05/2020

Electronic publication
