# Supplementary material for: Metformin delays neurological symptom onset in a mouse model of neuronal complex I deficiency
Source: JCI Insight. 2020 Nov 5;5(21):e141183. doi: 10.1172/jci.insight.141183 (PMC7710273; doi:10.1172/jci.insight.141183)
Supplement: supplemental data [file jciinsight-5-141183-s108.pdf]

Supplementary Methods

Metabolon Platform

Sample Accessioning: Following receipt, samples were inventoried and immediately stored at -80°C. Each sample received was accessioned into the Metabolon LIMS system and was assigned by the LIMS a unique identifier that was associated with the original source identifier only. This identifier was used to track all sample handling, tasks, results, etc. The samples (and all derived aliquots) were tracked by the LIMS system. All portions of any sample were automatically assigned their own unique identifiers by the LIMS when a new task was created; the relationship of these samples was also tracked. All samples were maintained at -80°C until processed.

Sample Preparation: Samples were prepared using the automated MicroLab STAR® system from Hamilton Company. Several recovery standards were added prior to the first step in the extraction process for QC purposes. To remove protein, dissociate small molecules bound to protein or trapped in the precipitated protein matrix, and to recover chemically diverse metabolites, proteins were precipitated with methanol under vigorous shaking for 2 min (Glen Mills GenoGrinder 2000) followed by centrifugation. The resulting extract was divided into five fractions: two for analysis by two separate reverse phase (RP)/UPLC-MS/MS methods with positive ion mode electrospray ionization (ESI), one for analysis by RP/UPLC-MS/MS with negative ion mode ESI, one for analysis by HILIC/UPLC-MS/MS with negative ion mode ESI, and one sample was reserved for backup. Samples were placed briefly on a TurboVap® (Zymark) to remove the organic solvent. The sample extracts were stored overnight under nitrogen before preparation for analysis.

QA/QC: Several types of controls were analyzed in concert with the experimental samples: a pooled matrix sample generated by taking a small volume of each experimental sample (or alternatively, use of a pool of well-characterized human plasma) served as a technical replicate throughout the data set; extracted water samples served as process blanks; and a cocktail of QC standards that were carefully chosen not to interfere with the measurement of endogenous compounds were spiked into every analyzed sample, allowed instrument performance monitoring and aided chromatographic alignment. Tables 1 and 2 describe these QC samples and standards. Instrument variability was determined by calculating the median relative standard deviation (RSD) for the standards that were added to each sample prior to injection into the mass spectrometers. Overall process variability was determined by calculating the median RSD for all endogenous metabolites (i.e., non-instrument standards) present in 100% of the pooled matrix samples. Experimental samples were randomized across the platform run with QC samples spaced evenly among the injections, as outlined in Figure 1.

Table 1: Description of Metabolon QC Samples

Type	Description	Purpose
MTRX	Large pool of human plasma maintained by Metabolon that has been characterized extensively.	Assure that all aspects of the Metabolon process are operating within specifications.
CMTRX	Pool created by taking a small aliquot from every customer sample.	Assess the effect of a non-plasma matrix on the Metabolon process and distinguish biological variability from process variability.
PRCS	Aliquot of ultra-pure water	Process Blank used to assess the contribution to compound signals from the process.
SOLV	Aliquot of solvents used in extraction.	Solvent Blank used to segregate contamination sources in the extraction.

Table 2: Metabolon QC Standards

Type	Description	Purpose
RS	Recovery Standard	Assess variability and verify performance of extraction and instrumentation.
IS	Internal Standard	Assess variability and performance of instrument.

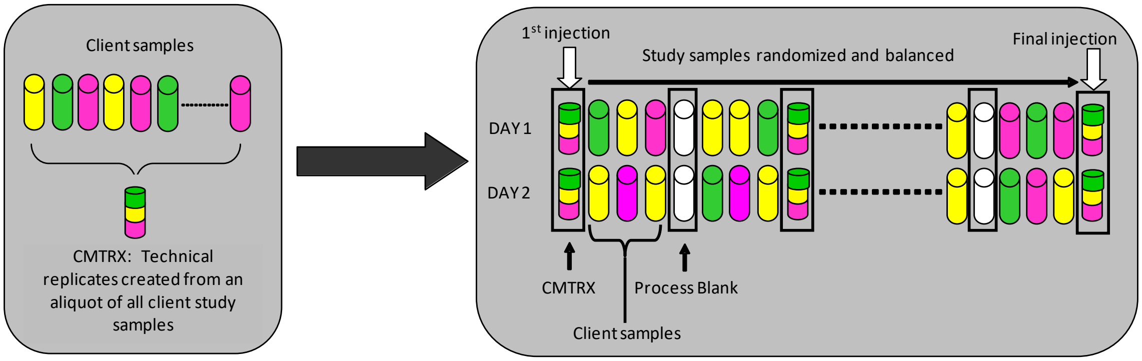

Figure 1. Preparation of client-specific technical replicates. A small aliquot of each client sample (colored cylinders) is pooled to create a CMTRX technical replicate sample (multi-colored cylinder), which is then injected periodically throughout the platform run. Variability among consistently detected biochemicals can be used to calculate an estimate of overall process and platform variability.

Ultrahigh Performance Liquid Chromatography-Tandem Mass Spectroscopy (UPLC-MS/MS): All methods utilized a Waters ACQUITY ultra-performance liquid chromatography (UPLC) and a Thermo Scientific Q-Exactive high resolution/accurate mass spectrometer interfaced with a heated electrospray ionization (HESI-II) source and Orbitrap mass analyzer operated at 35,000 mass resolution. The sample extract was dried then reconstituted in solvents compatible to each of the four methods. Each reconstitution solvent contained a series of standards at fixed concentrations to ensure injection and chromatographic consistency. One aliquot was analyzed using acidic positive ion conditions, chromatographically optimized for more hydrophilic compounds. In this method, the extract was gradient eluted from a C18 column (Waters UPLC BEH C18-2.1x100 mm, 1.7 µm) using water and methanol, containing 0.05% perfluoropentanoic acid (PFPA) and 0.1% formic acid (FA). Another aliquot was also analyzed using acidic positive ion conditions, however it was chromatographically optimized for more hydrophobic compounds. In this method, the extract was gradient eluted from the same afore mentioned C18 column using methanol, acetonitrile, water, 0.05% PFPA and 0.01% FA and was operated at an overall higher organic content. Another aliquot was analyzed using basic negative ion optimized conditions using a separate dedicated C18 column. The basic extracts were gradient eluted from the column using methanol and water, however with 6.5mM Ammonium Bicarbonate at pH 8. The fourth aliquot was analyzed via negative ionization following elution from a HILIC column (Waters UPLC BEH Amide 2.1x150 mm, 1.7 µm) using a gradient consisting of water and acetonitrile with 10mM Ammonium Formate, pH 10.8. The MS analysis alternated between MS and data-dependent MS^n scans using dynamic exclusion. The scan range varied slighted between methods but covered 70-1000 m/z. Raw data files are archived and extracted as described below.

Bioinformatics: The informatics system consisted of four major components, the Laboratory Information Management System (LIMS), the data extraction and peak-identification software, data processing tools for QC and compound identification, and a collection of information interpretation and visualization tools for use by data analysts. The hardware and software foundations for these informatics components were the LAN backbone, and a database server running Oracle 10.2.0.1 Enterprise Edition.

LIMS: The purpose of the Metabolon LIMS system was to enable fully auditable laboratory automation through a secure, easy to use, and highly specialized system. The scope of the Metabolon LIMS system encompasses sample accessioning, sample preparation and instrumental analysis and reporting and advanced data analysis. All of the subsequent software systems are grounded in the LIMS data structures. It has been modified to leverage and interface with the in-house information extraction and data visualization systems, as well as third party instrumentation and data analysis software.

Data Extraction and Compound Identification: Raw data was extracted, peak-identified and QC processed using Metabolon's hardware and software. These systems are built on a web-service platform utilizing Microsoft's .NET technologies, which run on high-performance application servers and fiber-channel storage arrays in clusters to provide active failover and load-balancing. Compounds were identified by comparison to library entries of purified standards or recurrent unknown entities. Metabolon maintains a library based on authenticated standards that

contains the retention time/index (RI), mass to charge ratio (*m/z)*, and chromatographic data (including MS/MS spectral data) on all molecules present in the library. Furthermore, biochemical identifications are based on three criteria: retention index within a narrow RI window of the proposed identification, accurate mass match to the library +/- 10 ppm, and the MS/MS forward and reverse scores between the experimental data and authentic standards. The MS/MS scores are based on a comparison of the ions present in the experimental spectrum to the ions present in the library spectrum. While there may be similarities between these molecules based on one of these factors, the use of all three data points can be utilized to distinguish and differentiate biochemicals. More than 3300 commercially available purified standard compounds have been acquired and registered into LIMS for analysis on all platforms for determination of their analytical characteristics. Additional mass spectral entries have been created for structurally unnamed biochemicals, which have been identified by virtue of their recurrent nature (both chromatographic and mass spectral). These compounds have the potential to be identified by future acquisition of a matching purified standard or by classical structural analysis.

Curation: A variety of curation procedures were carried out to ensure that a high quality data set was made available for statistical analysis and data interpretation. The QC and curation processes were designed to ensure accurate and consistent identification of true chemical entities, and to remove those representing system artifacts, mis-assignments, and background noise. Metabolon data analysts use proprietary visualization and interpretation software to confirm the consistency of peak identification among the various samples. Library matches for each compound were checked for each sample and corrected if necessary.

Metabolite Quantification and Data Normalization: Peaks were quantified using area-under-the-curve. For studies spanning multiple days, a data normalization step was performed to correct variation resulting from instrument inter-day tuning differences. Essentially, each compound was corrected in run-day blocks by registering the medians to equal one (1.00) and normalizing each data point proportionately (termed the "block correction"; Figure 2). For studies that did not require more than one day of analysis, no normalization is necessary, other than for purposes of data visualization. In certain instances, biochemical data may have been normalized to an additional factor (e.g., cell counts, total protein as determined by Bradford assay, osmolality, etc.) to account for differences in metabolite levels due to differences in the amount of material present in each sample.

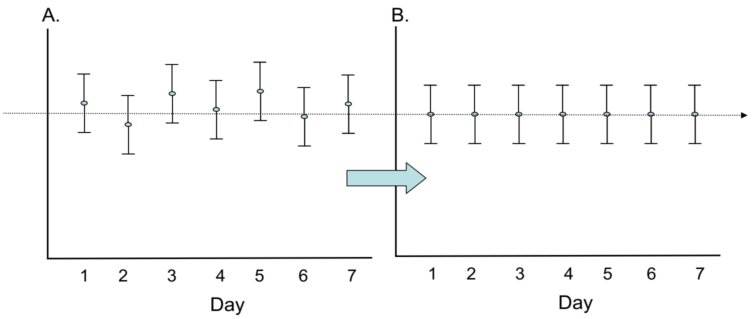

Figure 2: Visualization of data normalization steps for a multiday platform run.

Statistical Methods and Terminology

Statistical Calculations: For many studies, two types of statistical analysis are usually performed: (1) significance tests and (2) classification analysis. Standard statistical analyses are performed in ArrayStudio on log transformed data. For those analyses not standard in ArrayStudio, the programs R (http://cran.r-project.org/) or JMP are used. Below are examples of frequently employed significance tests and classification methods followed by a discussion of p- and q-value significance thresholds.

1. **Welch's two-sample *t*-test**

   Welch's two-sample *t*-test is used to test whether two unknown means are different from two independent populations.

   This version of the two-sample *t*-test allows for unequal variances (variance is the square of the standard deviation) and has an *approximate t*-distribution with degrees of freedom estimated using Satterthwaite's approximation. The test statistic is given by $t = (\bar{x}_1 - \bar{x}_2)/\sqrt{s_1^2/n_1 + s_2^2/n_2}$, and the degrees of freedom is given by $\left(\frac{s_1^2}{n_1} + \frac{s_2^2}{n_2}\right)^2 / \left(\frac{\left(\frac{s_1^2}{n_1}\right)^2}{n_1 - 1} + \frac{\left(\frac{s_2^2}{n_2}\right)^2}{n_2 - 1}\right)$, where $\bar{x}_1$, $\bar{x}_2$ are the sample means, s_1, s_2, are the sample standard deviations, and n_1, n_2 are the samples sizes from groups 1 and 2, respectively. We typically use a two-sided test (tests whether the means are different) as opposed to a one-sided test (tests whether one mean is greater than the other).

2. **p- values**

   For statistical significance testing, p-values are given. The lower the p-value, the more evidence we have that the null hypothesis (typically that two population means are equal) is not true. If "statistical significance" is declared for p-values less than 0.05, then 5% of the time we incorrectly conclude the means are different, when actually they are the same.

   The p-value is the probability that the test statistic is at least as extreme as observed in this experiment given that the null hypothesis is true. Hence, the more extreme the statistic, the lower the p-value and the more evidence the data gives against the null hypothesis.

3. **q-values**

   The level of 0.05 is the false positive rate when there is one test. However, for a large number of tests we need to account for false positives. There are different methods to correct for multiple testing. The oldest methods are family-wise error rate adjustments (Bonferroni, Tukey, etc.), but these tend to be extremely conservative for a very large number of tests. With gene arrays, using the False Discovery Rate (FDR) is more common.

The family-wise error rate adjustments give one a high degree of confidence that there are zero false discoveries. However, with FDR methods, one can allow for a small number of false discoveries. The FDR for a given set of compounds can be estimated using the q-value (see Storey J and Tibshirani R. (2003) Statistical significance for genomewide studies. Proc. Natl. Acad. Sci. USA 100: 9440-9445; PMID: 12883005).

In order to interpret the q-value, the data must first be sorted by the p-value then choose the cutoff for significance (typically $p<0.05$). The q-value gives the false discovery rate for the selected list (i.e., an estimate of the proportion of false discoveries for the list of compounds whose p-value is below the cutoff for significance). For Table 1 below, if the whole list is declared significant, then the false discovery rate is approximately 10%. If everything from Compound 079 and above is declared significant, then the false discovery rate is approximately 2.5%.

Table 1: Example of q-value interpretation

Compound	*p*-value	*q*-value
Compound 103	0.0002	0.0122
Compound 212	0.0004	0.0122
Compound 076	0.0004	0.0122
Compound 002	0.0005	0.0122
Compound 168	0.0006	0.0122
Compound 079	0.0016	0.0258
Compound 113	0.0052	0.0631
Compound 050	0.0053	0.0631
Compound 098	0.0061	0.0647
Compound 267	0.0098	0.0939

1. Random Forest

Random forest is a supervised classification technique based on an ensemble of decision trees (see Breiman L. (2001) Random Forests. Machine Learning. 45: 5-32; http://link.springer.com/article/10.1023%2FA%3A1010933404324). For a given decision tree, a random subset of the data with identifying true class information is selected to build the tree ("bootstrap sample" or "training set"), and then the remaining data, the "out-of-bag" (OOB) variables, are passed down the tree to obtain a class prediction for each sample. This process is repeated thousands of times to produce the forest. The final classification of each sample is determined by computing the class prediction frequency ("votes") for the OOB variables over the whole forest. For example, suppose the random forest consists of 50,000 trees and that 25,000 trees had a prediction for sample 1. Of these 25,000, suppose 15,000 trees classified the sample as belonging to Group A and the remaining 10,000 classified it as belonging to Group B. Then the votes are 0.6 for Group A and 0.4 for Group B, and hence the final classification is Group A. This method is unbiased since the prediction for each sample is based on trees built from a subset of samples that do not include that sample. When the full forest is grown, the class predictions are compared to the true classes, generating the "OOB error rate" as a measure of prediction accuracy. Thus, the prediction accuracy is an unbiased estimate of how well one can predict sample class in a new data set. Random forest has several advantages – it makes no parametric assumptions, variable selection is not needed, it

does not overfit, it is invariant to transformation, and it is fairly easy to implement with R.

To determine which variables (biochemicals) make the largest contribution to the classification, a "variable importance" measure is computed. We use the "Mean Decrease Accuracy" (MDA) as this metric. The MDA is determined by randomly permuting a variable, running the observed values through the trees, and then reassessing the prediction accuracy. If a variable is not important, then this procedure will have little change in the accuracy of the class prediction (permuting random noise will give random noise). By contrast, if a variable is important to the classification, the prediction accuracy will drop after such a permutation, which we record as the MDA. Thus, the random forest analysis provides an "importance" rank ordering of biochemicals; we typically output the top 30 biochemicals in the list as potentially worthy of further investigation.

4. **Hierarchical Clustering**

Hierarchical clustering is an unsupervised method for clustering the data, and can show large-scale differences. There are several types of hierarchical clustering and many distance metrics that can be used. A common method is complete clustering using the Euclidean distance, where each sample is a vector with all of the metabolite values. The differences seen in the cluster may be unrelated to the treatment groups or study design.

5. **Principal Components Analysis (PCA)**

Principal components analysis is an unsupervised analysis that reduces the dimension of the data. Each principal component is a linear combination of every metabolite and the principal components are uncorrelated. The number of principal components is equal to the number of observations.

The first principal component is computed by determining the coefficients of the metabolites that maximizes the variance of the linear combination. The second component finds the coefficients that maximize the variance with the condition that the second component is orthogonal to the first. The third component is orthogonal to the first two components and so on. The total variance is defined as the sum of the variances of the predicted values of each component (the variance is the square of the standard deviation), and for each component, the proportion of the total variance is computed. For example, if the standard deviation of the predicted values of the first principal component is 0.4 and the total variance = 1, then 100*0.4*0.4/1 = 16% of the total variance is explained by the first component. Since this is an unsupervised method, the main components may be unrelated to the treatment groups, and the "separation" does not give an estimate of the true predictive ability.

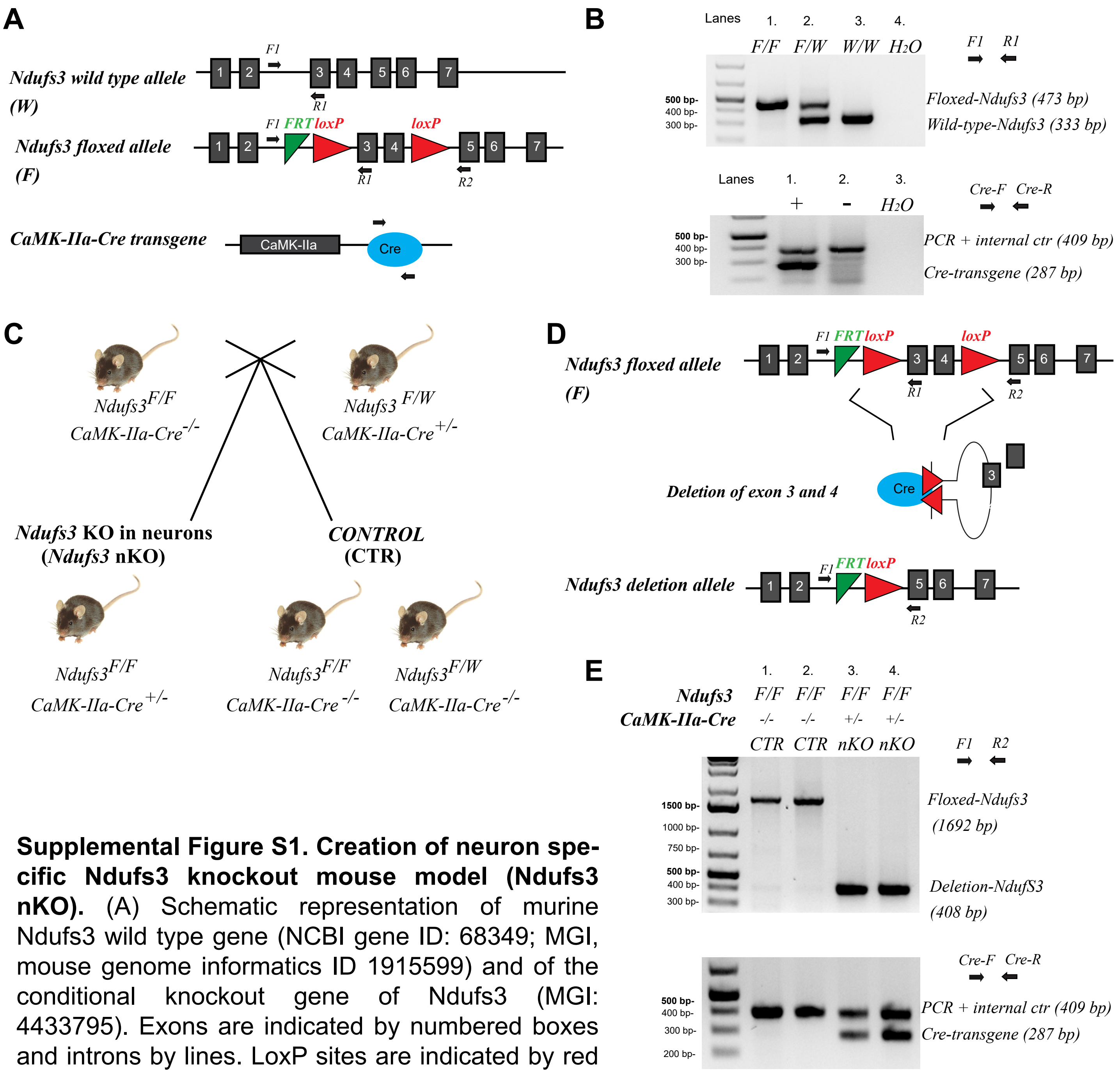

Supplemental Figure S1. Creation of neuron specific Ndufs3 knockout mouse model (Ndufs3 nKO). (A) Schematic representation of murine Ndufs3 wild type gene (NCBI gene ID: 68349; MGI, mouse genome informatics ID 1915599) and of the conditional knockout gene of Ndufs3 (MGI: 4433795). Exons are indicated by numbered boxes and introns by lines. LoxP sites are indicated by red triangles, and FRT sequence by green triangle. (B) Screening of wild type and conditional (floxed) Ndufs3 genes and the CaMKIIα-Cre transgene in the transgenic mice. PCR products from genomic DNA of mouse tails were amplified with primers F1 and R1 (showed in A) for NdfuS3 gene and with primers Cre-F and Cre-R (showed in C) for the detection of the CaMKIIα-Cre transgene. (C) Crossing scheme used to generate neuron specific Ndufs3 KO mouse model, named Ndufs3 nKO mice. Ndufs3 knockout mice are homozygous for the floxed Ndufs3 gene and positive for the CaMKIIa-Cre transgene. (D) Schematic representation of the deletion of exons 3 and 4 in Ndfus3 gene after Cre recombinase is expressed. (E) Molecular confirmation of the deletion of exons 3 and 4 of Ndufs3 gene in cortices of the nKO mice. PCR products from genomic DNA of cortices were amplified with primers F1 and R2 (showed in C). Upper panel: floxed NdufS3 gene (1692 bp) and deleted Ndufs3 (408 bp) Lower panel: Cre-transgene (287 bp) and internal positive control (409 bp) gene.

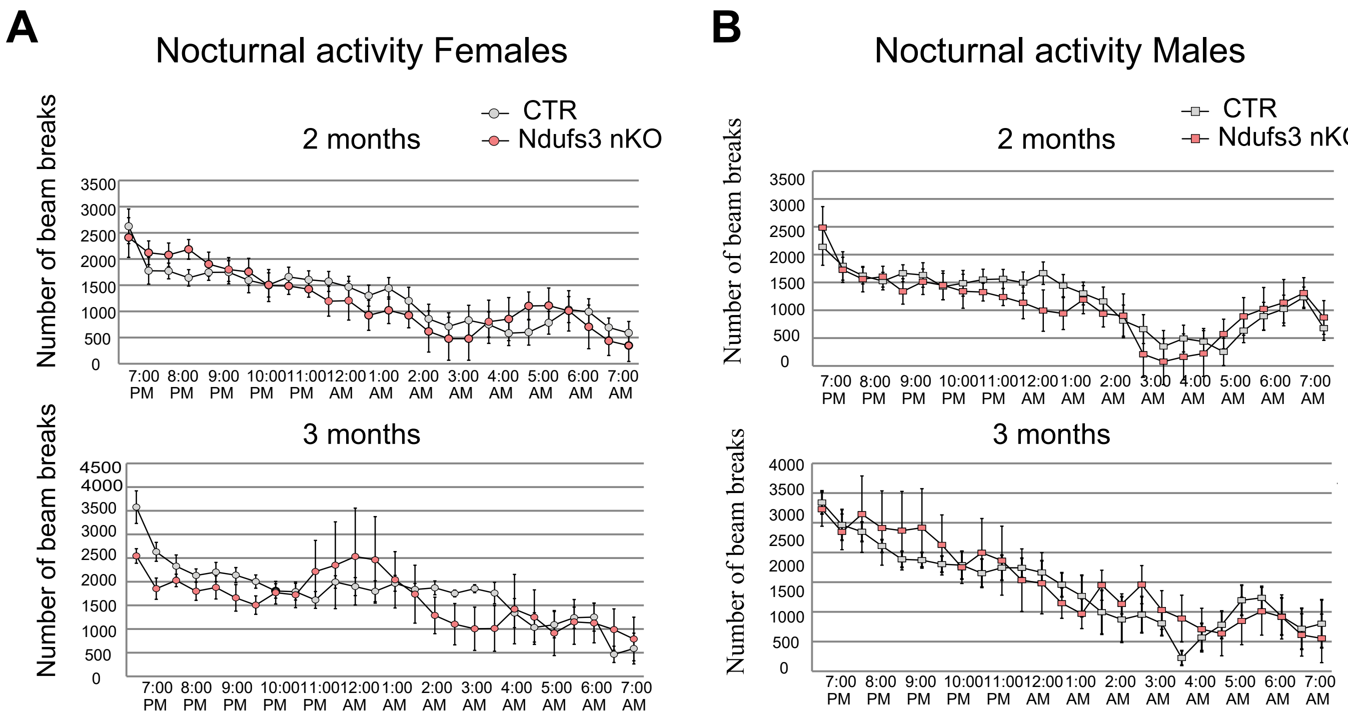

Supplemental Figure S2. Phenotypic characterization of Ndufs3 nKO animals. Nocturnal ambulatory activity of females (A) and males (B) Ndufs3 nKO and aged matched control littermates of 2 and 3 months old mice. (n=4-6/group), P values were calculated by Student's t test.

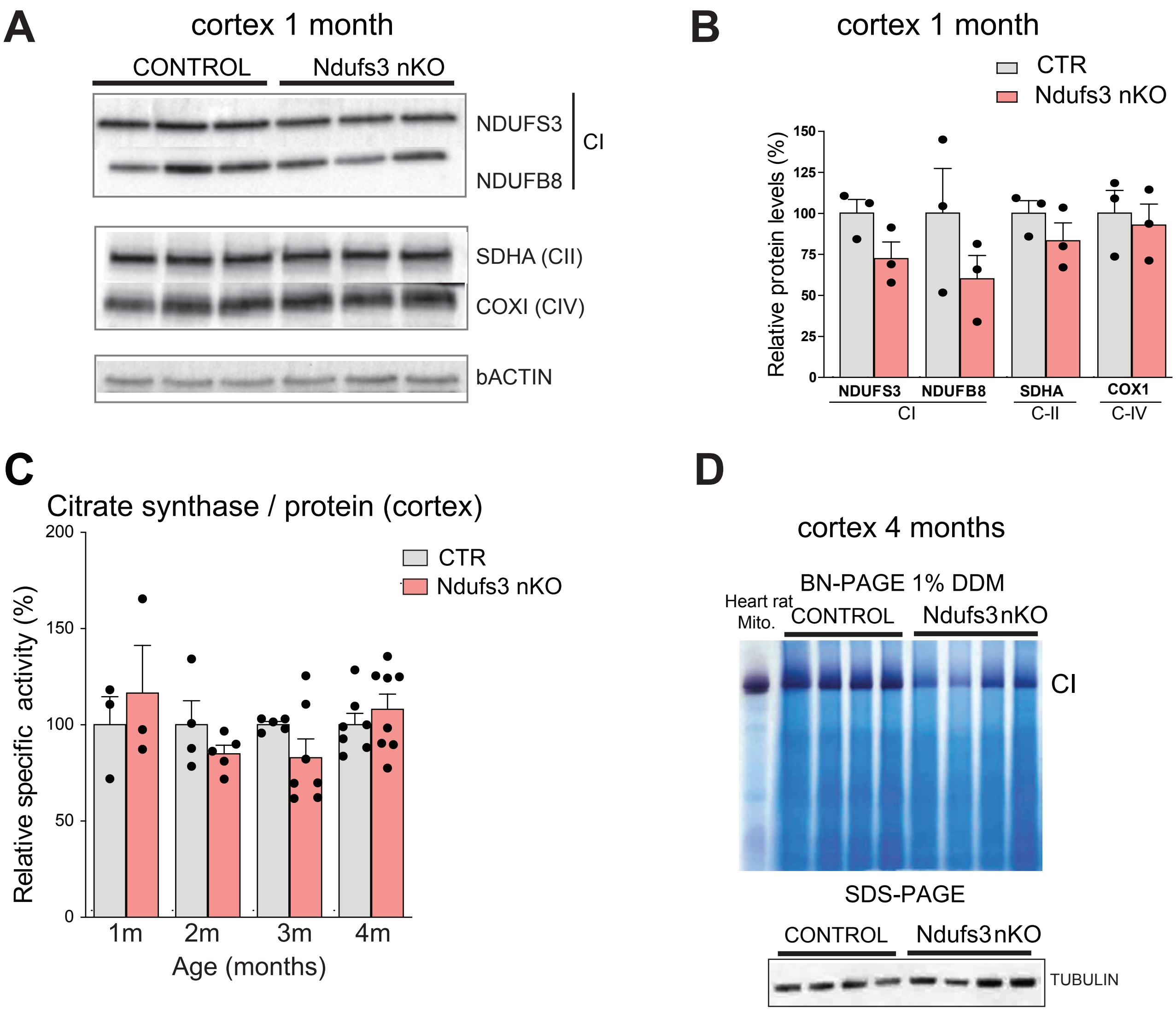

Supplemental Figure S3. Mitochondrial protein quantification in cortex homogenates from 1-month-old animals and Citrate synthase activity of cortex homogenates of Ndufs3 nKO animals at different ages. (A) Western blots of cortex homogenates of CTR and Ndufs3 nKO animals at 1 month, using antibodies against NDUFS3, NDUFB8 (complex I subunits), SDHA (complex II subunit), COX1 (complex IV subunit), and bACTIN. (B) Quantification of the western blots in panel A. Bars represent means ±SEM (n=3/group). (C) Spectrophotometric Citrate synthase normalized to protein content measured in cortex homogenates from 1, 2, 3, and 4 months old mice. Bars represent means ±SEM (n=4-5/group). Citrate synthase activity in cortex homogenates from Ndufs3 nKO was similar to control mice at all ages tested. P values were determined by Student's t test. (D) BN-PAGE In-Gel Activity of Complex I in homogenates from cortex of wild-type and Ndufs3 nKO animals at 4 months. Error bars represent SEM.

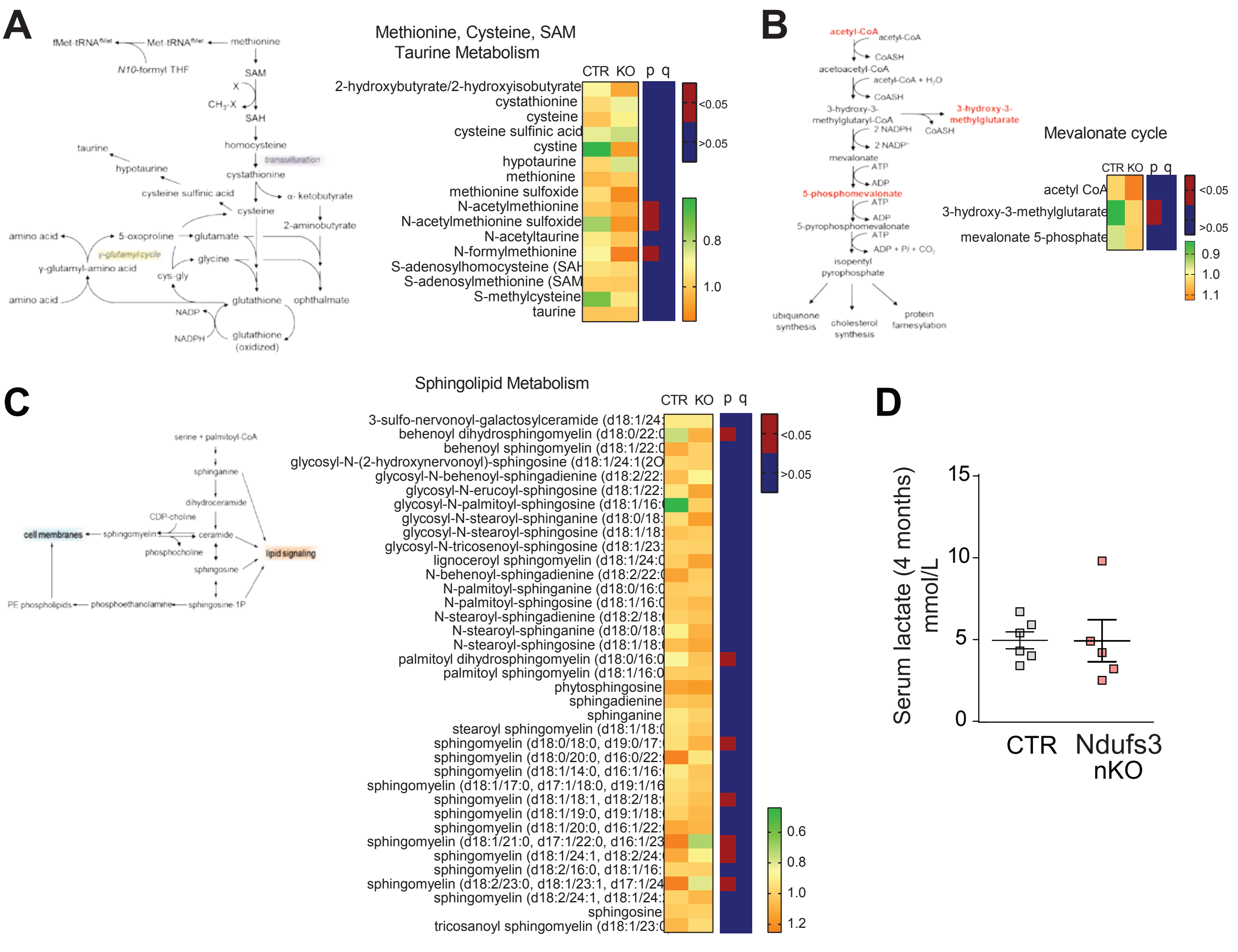

Supplemental Figure S4. Methionine, Mevalonate and Sphingolipid metabolites in Ndufs3 nKO brains. Cortex from 2.5 months old Ndufs3 nKO male mice compared to control littermates were used for the analysis. (n=6/group). Groups were compared using Welch's Two Sample t-Test and statistical significance is declared for p-values < 0.05. q-value significance was determined from the 503 significant hits with p-value <0.05). Orange boxes represent increased value in Ndufs3 nKO compared to controls, green boxes represent decreased value in Ndufs3 nKO compared to controls. (A) Scheme of the Methionine related metabolism pathway (B) Mevalonate related metabolism pathway and (C) Sphingolipid related metabolism pathway. Group average heatmap is shown for each pathway related metabolites. (D) Serum lactate levels measured in plasma from 4-months old mice. N=6 control group, and n= 8 Ndufs3 nKO. Data represent means ±SEM.

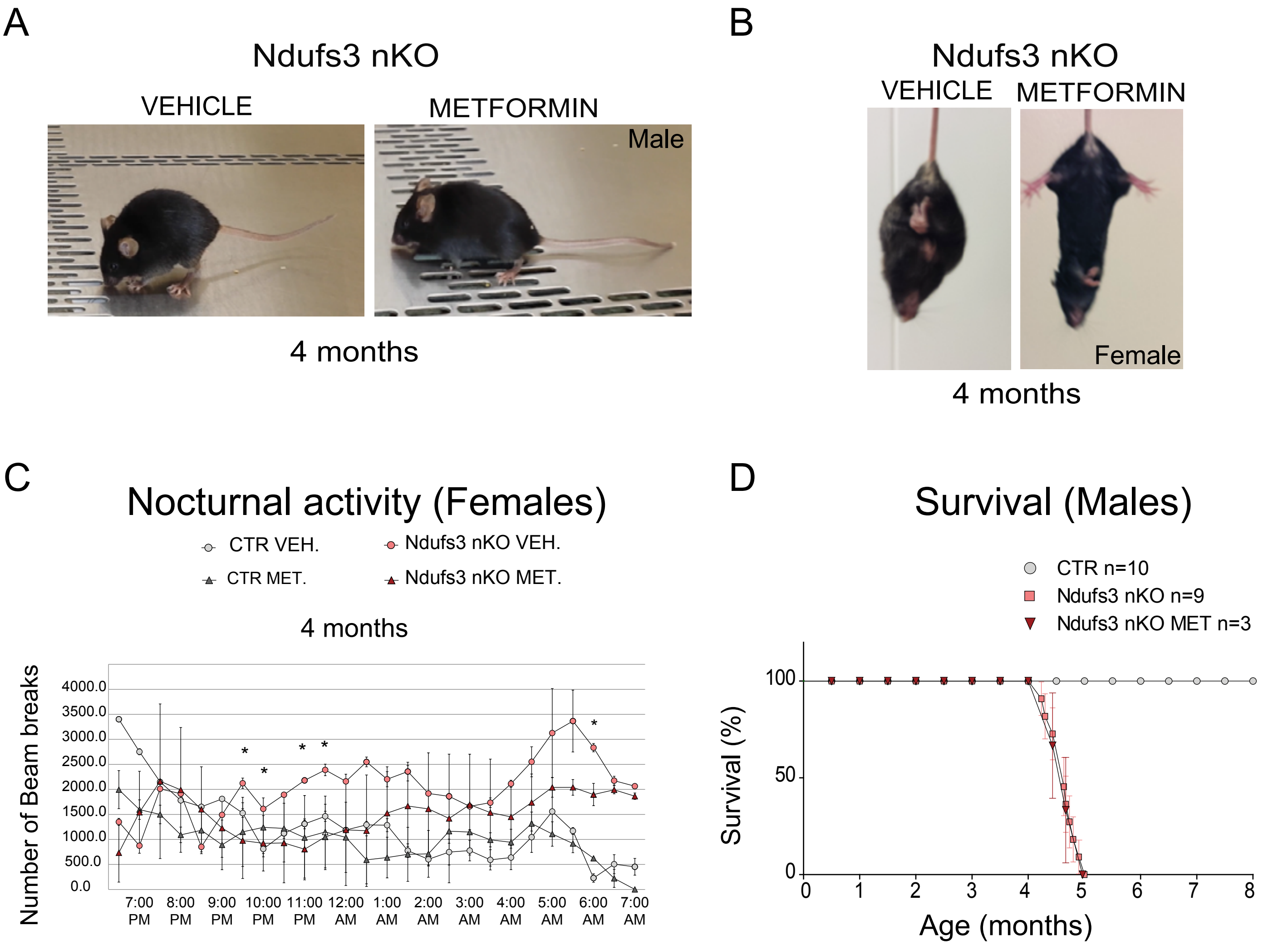

Supplemental Figure S5. Metformin treatment in Ndfus3 nKO animals. (A) Appearance of vehicle-treated and metformin-treated 4 months old Ndfus3 nKO females. (B) Representative image of a tail suspension test of vehicle-treated and metformin-treated 4 months old Ndfus3 nKO females. (C) Nocturnal ambulatory activity of 4 months old Ndufs3 nKO and aged matched control females. Statistical significance was determined using one-way ANOVA. Pair-wise Bonferroni post-test was used to compare different groups in all panels. (D) Survival curve of control males (grey circles), Ndufs3 nKO male mice (pink squares), and metformin treated Ndufs3 nKO male mice (red triangles).

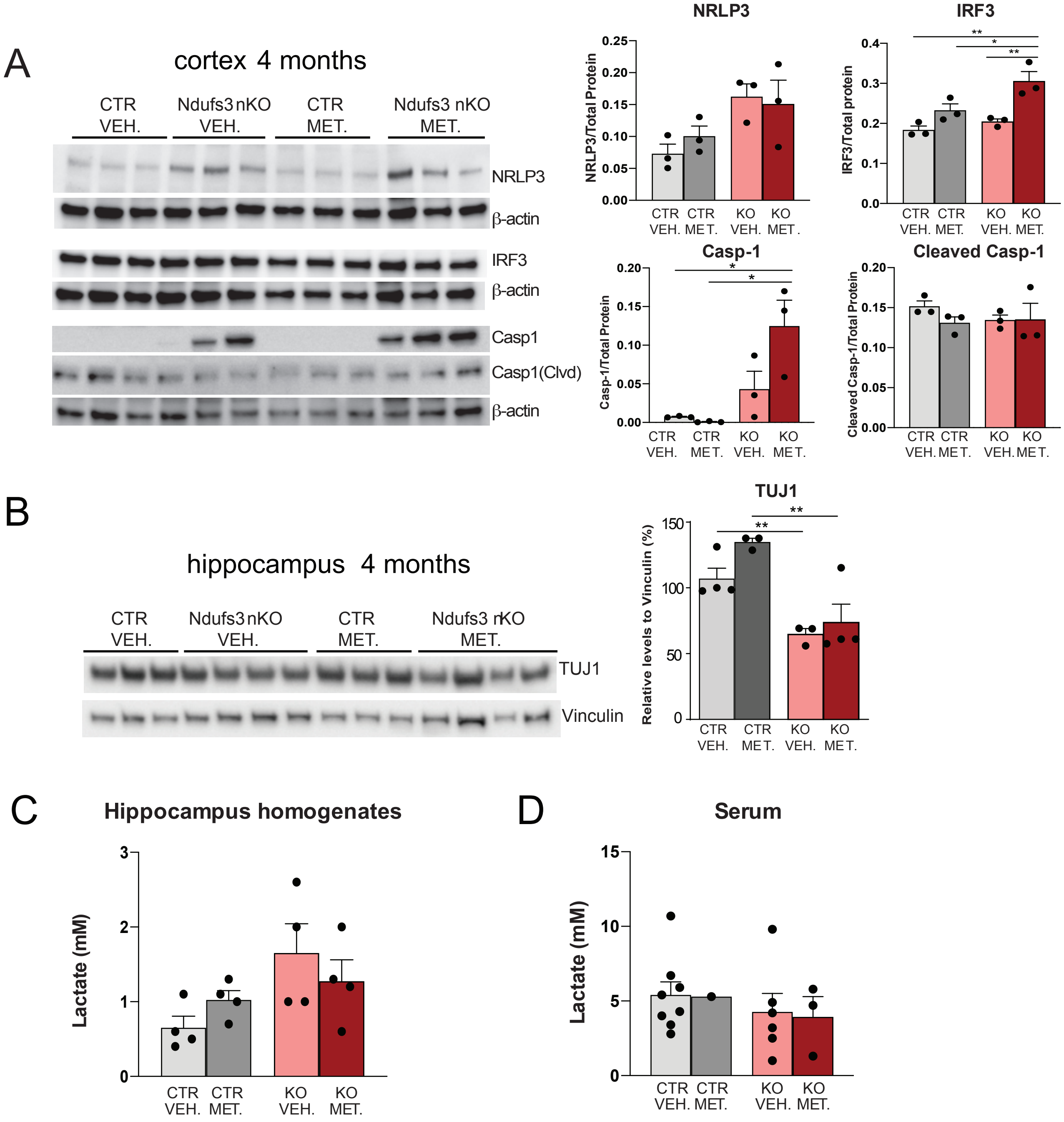

Supplemental Figure S6. TUJ1, inflammatory markers and lactate levels in 4 month-old animals. (A) Western blots and relative quantification for inflammatory markers of cortex homogenates of vehicle-treated controls and Ndufs3 nKO animals and metformin-treated controls and Ndufs3 nKO animals at 4 months. (B) Western blots probing TUJ1 and relative quantification of hippocampus homogenates of vehicle-treated controls and Ndufs3 nKO animals and metformin-treated controls and Ndufs3 nKO animals at 4 months. (C-D) Lactate levels were determined in hippocampus homogenates (C) and in serum (D). No changes associated with metformin were detected. Bars represent means ±SEM. n=3/group. P values were determined by ANOVA.

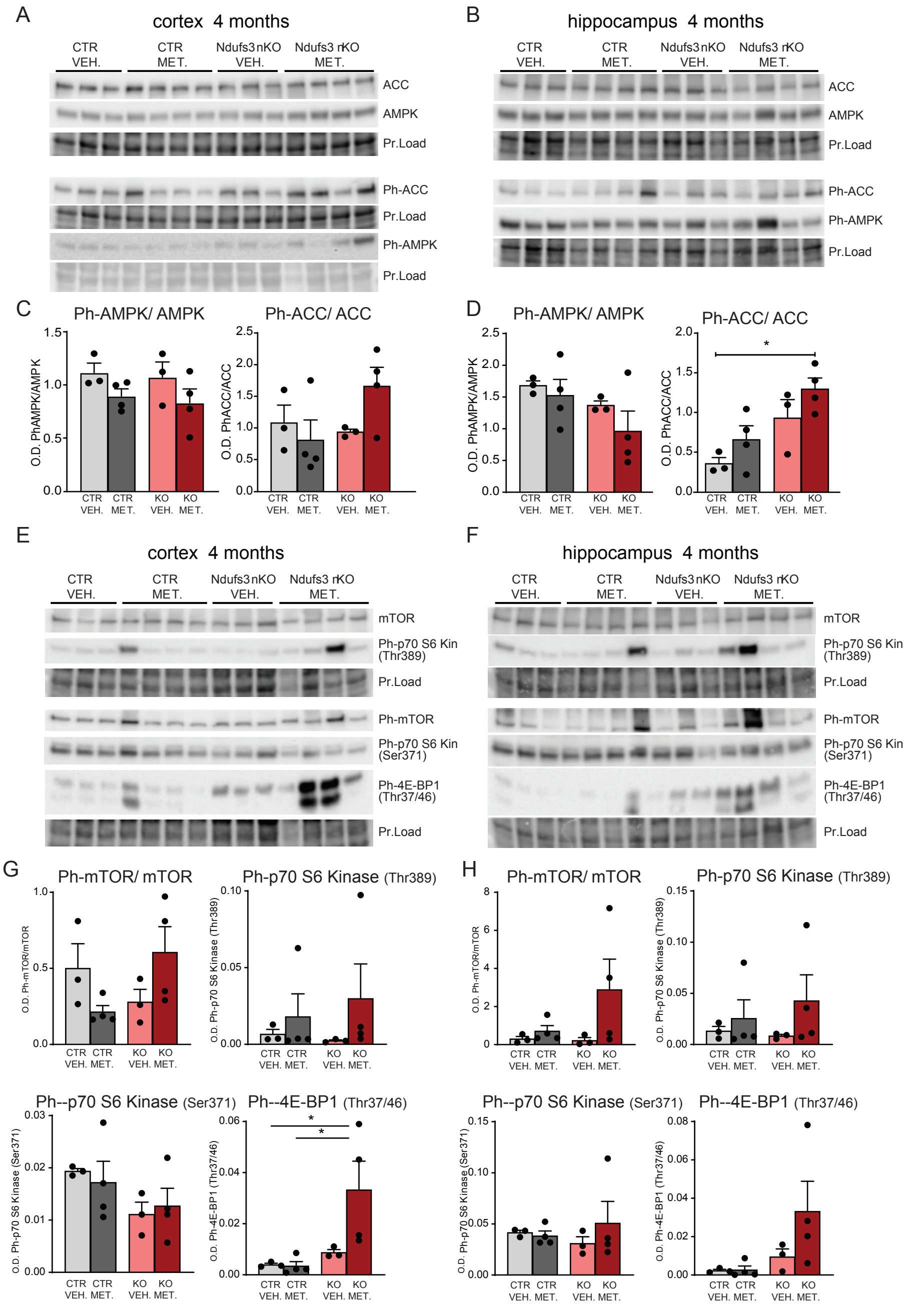

Supplemental Figure S7. Effects of metformin on mTOR-dependent phosphorylation in cortex and hippocampus from Ndufs3 nKO mice of 4 months old. (A-B) Levels of total AMPK, p-AMPK (thr 172), ACC, p-ACC (the product of AMPK-dependent phosphorylation of ACC at Ser79) were assayed by western blotting on cortex and hippocampus regions of 4-month-old controls and Ndufs3 nKO females treated with vehicle or metformin. (C-D). Relative quantifications of protein levels determined in A and B sections. (E-F) Levels of , p-S6 -Thr 389, p-S6 -Ser 371- and p-4E-BP1-thr37/46- (the product of mTOR-dependent phosphorylation) and mTOR were measured by western blotting on cortex and hippocampus regions of 4-month-old controls and Ndufs3 nKO females treated with vehicle or metformin. (G-H). Relative quantifications of protein levels determined in A and B sections. Bars represent means ±SEM of n=3-4 for each group. P values were determined by one-way ANOVA followed by a Bonferroni post-hoc comparison. Significance between groups is indicated as *p<0.05, **p<0.01, ***p<0.001.
